# Three‐year quantitative magnetic resonance imaging and phosphorus magnetic resonance spectroscopy study in lower limb muscle in dysferlinopathy

**DOI:** 10.1002/jcsm.12987

**Published:** 2022-04-03

**Authors:** Harmen Reyngoudt, Fiona E. Smith, Ericky Caldas de Almeida Araújo, Ian Wilson, Roberto Fernández‐Torrón, Meredith K. James, Ursula R. Moore, Jordi Díaz‐Manera, Benjamin Marty, Noura Azzabou, Heather Gordish, Laura Rufibach, Tim Hodgson, Dorothy Wallace, Louise Ward, Jean‐Marc Boisserie, Julien Le Louër, Heather Hilsden, Helen Sutherland, Aurélie Canal, Jean‐Yves Hogrel, Marni Jacobs, Tanya Stojkovic, Kate Bushby, Anna Mayhew, Jain Clinical Outcome Study for Dysferlinopathy consortium, Volker Straub, Pierre G. Carlier, Andrew M. Blamire

**Affiliations:** ^1^ NMR Laboratory Neuromuscular Investigation Center, Institute of Myology Paris France; ^2^ NMR Laboratory CEA/DRF/IBFJ/MIRCen Paris France; ^3^ Magnetic Resonance Centre, Translational and Clinical Research Institute, Faculty of Medical Sciences Newcastle University Newcastle upon Tyne UK; ^4^ The John Walton Muscular Dystrophy Research Centre, Translational and Clinical Research Institute Newcastle University and Newcastle Hospitals NHS Foundation Trust Newcastle upon Tyne UK; ^5^ Neuromuscular Area, Biodonostia Health Research Institute, Neurology Service Donostia University Hospital Donostia‐San Sebastian Spain; ^6^ Neuromuscular Disorders Unit, Neurology Department Hospital Santa Creu i Sant Pau Barcelona Spain; ^7^ Centro de Investigación Biomédica en Red en Enfermedades Raras (CIBERER) Valencia Spain; ^8^ Center for Translational Science, Division of Biostatistics and Study Methodology Children's National Health System Washington DC USA; ^9^ Pediatrics, Epidemiology and Biostatistics George Washington University Washington DC USA; ^10^ The JAIN Foundation Seattle WA USA; ^11^ Neuromuscular Physiology and Evaluation Laboratory Neuromuscular Investigation Center, Institute of Myology Paris France; ^12^ Neuromuscular Reference Center, Institute of Myology Pitié‐Salpêtrière Hospital (AP‐HP) Paris France; ^13^ University Paris‐Saclay, CEA, DRF, SHFJ Orsay France

**Keywords:** Dysferlinopathy, Quantitative MRI, ^31^P MRS, Longitudinal, Outcome measures

## Abstract

**Background:**

Natural history studies in neuromuscular disorders are vital to understand the disease evolution and to find sensitive outcome measures. We performed a longitudinal assessment of quantitative magnetic resonance imaging (MRI) and phosphorus magnetic resonance spectroscopy (^31^P MRS) outcome measures and evaluated their relationship with function in lower limb skeletal muscle of dysferlinopathy patients.

**Methods:**

Quantitative MRI/^31^P MRS data were obtained at 3 T in two different sites in 54 patients and 12 controls, at baseline, and three annual follow‐up visits. Fat fraction (FF), contractile cross‐sectional area (cCSA), and muscle water T_2_ in both global leg and thigh segments and individual muscles and ^31^P MRS indices in the anterior leg compartment were assessed. Analysis included comparisons between patients and controls, assessments of annual changes using a linear mixed model, standardized response means (SRM), and correlations between MRI and ^31^P MRS markers and functional markers.

**Results:**

Posterior muscles in thigh and leg showed the highest FF values. FF at baseline was highly heterogeneous across patients. In ambulant patients, median annual increases in global thigh and leg segment FF values were 4.1% and 3.0%, respectively (*P* < 0.001). After 3 years, global thigh and leg FF increases were 9.6% and 8.4%, respectively (*P* < 0.001). SRM values for global thigh FF were over 0.8 for all years. Vastus lateralis muscle showed the highest SRM values across all time points. cCSA decreased significantly after 3 years with median values of 11.0% and 12.8% in global thigh and global leg, respectively (*P* < 0.001). Water T_2_ values in ambulant patients were significantly increased, as compared with control values (*P* < 0.001). The highest water T_2_ values were found in the anterior part of thigh and leg. Almost all ^31^P MRS indices were significantly different in patients as compared with controls (*P* < 0.006), except for pH_w_, and remained, similar as to water T_2_, abnormal for the whole study duration. Global thigh water T_2_ at baseline was significantly correlated to the change in FF after 3 years (*ρ* = 0.52, *P* < 0.001). There was also a significant relationship between the change in functional score and change in FF after 3 years in ambulant patients (*ρ* = −0.55, *P* = 0.010).

**Conclusions:**

This multi‐centre study has shown that quantitative MRI/^31^P MRS measurements in a heterogeneous group of dysferlinopathy patients can measure significant changes over the course of 3 years. These data can be used as reference values in view of future clinical trials in dysferlinopathy or comparisons with quantitative MRI/S data obtained in other limb‐girdle muscular dystrophy subtypes.

## Introduction

Dysferlinopathy is an autosomal recessive neuromuscular disorder caused by mutations in the dysferlin gene, *DYSF*, leading to a deficiency of functional dysferlin, a protein that is highly expressed in muscle and that is essential in membrane repair.^1^ Dysferlinopathy is characterized by an active inflammatory and degenerative process leading ultimately to muscle fibre necrosis and muscle replacement by fibrous and fatty tissue.^2^ Dysferlinopathy patients have been labelled with different phenotypes, most importantly limb‐girdle muscular dystrophy type R2 (LGMD R2 [formerly LGMD 2B]), and Miyoshi myopathy (MM).^3^ Clinical symptoms usually start between late teenage years and 30 years and are associated with elevated serum creatine kinase (CK) levels.^1^, ^3^ The rate of disease progression is variable and is generally faster when the disease begins during early teenage years.^4^ Whereas skeletal muscle is highly affected, impacting daily life activity, involvement of cardiac muscle is uncommon in dysferlinopathy.^4^

In the last 10 years, magnetic resonance imaging (MRI) has been used as an outcome measure in several studies of dysferlinopathy patients.^5^, ^6^, ^7^, ^8^, ^9^ The largest of these studies was the Clinical Outcome Study (COS) for dysferlinopathy initiated by the Jain Foundation, which involved a worldwide multi‐centre natural history study in over 200 patients including clinical, biochemical, functional, strength, and imaging evaluations.^8^, ^10^ All these aforementioned studies described the use of T_1_‐weighted and/or T_2_‐weighted qualitative MRI. However, quantitative MRI has been increasingly applied in studies of neuromuscular diseases,^11^ including Duchenne muscular dystrophy (DMD),^12^, ^13^, ^14^, ^15^ Becker muscular dystrophy,^16^, ^17^ facioscapulohumeral dystrophy (FSHD),^18^ LGMD R9,^19^ inclusion body myositis,^20^ GNE myopathy,^21^ and late‐onset Pompe disease.^22^, ^23^

The objective of this study was to analyse the quantitative MRI and phosphorus magnetic resonance spectroscopy (^31^P MRS) data, acquired in the patients from the Jain COS study, both cross‐sectionally and longitudinally. Additionally, correlations between MRI and ^31^P MRS parameters and functional and strength data were investigated.

## Materials and methods

### Study set‐up and subjects

Quantitative MRI and ^31^P MRS data were obtained annually (between November 2012 and November 2017) for 3 years in 54 patients from two sites: Newcastle, UK (*n* = 42), and Paris, France (*n* = 12). These 54 subjects were a sub‐cohort of the 201 patients enrolled across 15 sites worldwide in the Jain COS for dysferlinopathy (Supporting Information, *Table* S1).^8^ Patients were included in the global study based on genetic confirmation for dysferlinopathy.^10^ Ambulatory status was defined as the ability to walk 10 m with or without walking aids or orthotics.^8^, ^10^ None of the patients were under corticosteroid treatment since at least 6 months before the start of the study. Data were also obtained in 12 age‐matched and sex‐matched healthy subjects. Results on the 1‐year fat fraction (FF) progression in the 12 patients from Paris have previously been reported.^24^

Written informed consent was obtained from patients prior to inclusion, in accordance with the 1964 Declaration of Helsinki and its later amendments. The study registration number was NCT01676077. Healthy control subjects were scanned as part of a methodology MRI/S protocol approved by local ethics committees (Newcastle: United Kingdom National Research Ethics Committee 1417/375/2017; Paris: CPP‐Ile de France VI—Groupe Hospitalier Pitié‐Salpêtrière, ID RCB: 2012‐A01689‐34). Informed consent was obtained from all controls.

### Quantitative magnetic resonance imaging and phosphorus magnetic resonance spectroscopy data acquisition

Data were acquired on one of two 3 T MRI clinical scanners: an Achieva (Philips Healthcare, Amsterdam, Netherlands) in Newcastle and Trio/Prisma (Siemens Healthineers, Erlangen, Germany) in Paris. For MRI, the local system's body RF coil for signal transmission and surface‐array coils for signal transmission were used. For ^31^P MRS, a flexible ^1^H/^31^P surface RF coil was employed (Newcastle: 14 cm, Philips; Paris: 11 cm, Rapid Biomedical GmbH, Rimpar, Germany). Patients were positioned feet‐first supine and all MRI sequences were centred at one‐third of the femur from the superior border of the patella and at the widest part of the calf.

Quantitative water‐fat imaging was performed using a 2D (Newcastle) or 3D (Paris) gradient echo 3‐point Dixon sequence. In Newcastle, Dixon scans were acquired in five 10 mm slices with a 20 mm interslice gap, covering a volume of 130 mm with an in‐plane resolution of 1.5 × 1.5 mm^2^, a flip angle of 8°, and with repetition time (TR)/echo times (TEs) = 10/3.45–4.60–5.75 ms. In Paris, the Dixon sequence was acquired in a 3D volume of 5 mm slices covering at least 170 mm with an in‐plane resolution of 1.0 × 1.0 mm^2^, a flip angle of 3°, and with TR/TEs = 10/2.75–3.95–5.15 ms. For water T_2_ mapping, a 2D multi‐spin‐echo (MSE) sequence was employed covering the 130 mm 2D Dixon volume in Newcastle (in‐plane resolution = 1.0 × 1.0 mm^2^) and corresponding to the 3D Dixon volume in Paris (in‐plane resolution = 1.4 × 1.4 mm^2^), with 17 equidistant echoes (TE_1_/ΔTE = 9.9 ms in Newcastle; TE_1_/ΔTE = 9.5 ms in Paris), a TR of 3000 ms, a slice thickness of 10 mm, and a slice gap of 30 mm.^25^ For calculating the transmit field (B_1_^+^) in each voxel, a B_1_ map sequence was run covering the same slices as the MSE sequence.^26^ ^31^P MRS data were obtained in the anterior part of the right leg, unless the muscles were completely replaced by fat, from a non‐localized free‐induction decay of 64 averages (a TR of 4000 ms, a bandwidth of 3000 Hz, 2048 data points). Total acquisition time for quantitative MRI and ^31^P MRS was approximately 40 min.

### Magnetic resonance imaging and phosphorus magnetic resonance spectroscopy data processing

All quantitative MRI data were processed using in‐house written Matlab (MathWorks, Natick, MA, USA) or Python (www.python.org) code (Newcastle, UK, and Paris, France, respectively).

Regions of interest (ROIs) were drawn manually on the shortest TE‐image of the MSE series, using the free software tool (www.itksnap.org), both left and right sides, in seven leg muscles (extensor digitorum longus, tibialis anterior, tibialis posterior, peroneus longus, soleus, gastrocnemius medialis/lateralis) and nine thigh muscles (vastus lateralis/intermedius/medialis, gracilis, sartorius, adductor magnus, biceps femoris long head, semimembranosus, semitendinosus), for the five central slices of the MSE scan. To determine FF and cross‐sectional area (CSA), the boundaries of the ROIs were drawn (by I. W.) following individual muscle delineation, avoiding inclusion of other muscles, subcutaneous and intermuscular fat, tendons, and major blood vessels. To assess water T_2_, ROIs delineated the interior of the muscle, avoiding visible fasciae and blood vessels (by J. L. L.).^25^ A third observer (R. F. T.) compared the two different segmentations and instructed the two observers to correct where necessary.

Fat fraction and CSA values were computed using the Dixon images (co‐registered to the five MSE image slices). Dixon data were reconstructed using a six‐component lipid model and considering a single T_2_* decay.^27^, ^28^ FF was calculated as SI (fat)/((SI)fat + (SI)water)*100 (with SI = signal intensity). FF maps that included subcutaneous and bone FF values < 95%, or partial fat–water swaps, were excluded for analysis. Before extracting FF and CSA values from the ROIs, FF maps were resized because of the difference in voxel size between Dixon and MSE images. When necessary, ROIs applied to the FF maps were trimmed or shifted due to overlapping subcutaneous fat or patient movement between Dixon and MSE acquisitions, respectively.

Using the MSE images, quantitative water T_2_ maps were reconstructed based on a tri‐exponential fitting procedure.^25^ Only pixels where B_1_^+^ values were between 80% and 120% of the prescribed flip angle were accepted for analysis. ROIs that included <10 pixels were excluded for analysis.

Contractile cross‐sectional area (cCSA), defined as lean muscle CSA (expressed in cm^2^) corresponding to the muscle volume fraction containing the contractile apparatus, was calculated using the FF and CSA values (cCSA = CSA*(1 − (0.01*FF))).^12^ We evaluated the global segment for cCSA, which corresponds to the sum of the individual muscle cCSA values, per segment. For the global values for thigh and leg FF and water T_2_, a weighted mean of the values as determined in the individual muscles was calculated.^24^ To evaluate disease progression, the annual and 3‐year changes in FF, ΔFF% (expressed in %, absolute difference), and cCSA, ΔcCSA_rel_ (expressed in %, relative difference compared with the precedent cCSA value), were evaluated.^21^ All MRI outcome measures are reported as mean values of all pixels in the ROI averaged over the five slices.

Phosphorus magnetic resonance spectroscopy data were processed as previously described,^13^, ^21^ using AMARES in jMRUI^29^ and Topspin (Bruker Medical GMbG, Ettlingen, Germany) to calculate P_i,tot_/PCr (total inorganic phosphate over phosphocreatine), P_i,b_/P_i,tot_ (alkaline P_i_ over P_i,tot_), P_i,tot_/γATP (P_i,tot_ over adenosine triphosphate), PCr/γATP, PDE/γATP (phosphodiesters over γATP), and PME/γATP (phosphomonoesters over γATP) ratios, as well as values of pH_w_ (i.e. weighted pH, based on the relative weights of cytosolic P_i_, P_i,a_, and alkaline P_i_, P_i,b_, resonances) and the intramuscular magnesium concentration, [Mg^2+^]. Only ^31^P MRS data with sufficient signal‐to‐noise ratio or SNR (i.e. >10 for PCr resonance) were accepted for final analysis.

Inter‐site variability between the Paris and Newcastle sites, assessed in four healthy control subjects scanned at both locations, was 1.2% (absolute difference) for FF, 1.0% (relative difference for cCSA), 0.5 ms for water T_2_, and 0.03 units for P_i,tot_/PCr and PDE/γATP. No inter‐site corrections were applied for any of the outcome measures.

### Functional, strength, and creatine kinase assessments

The dysferlinopathy‐adapted 29‐item scale North Star Assessment for LGMD‐type Dystrophies (NSAD) was used.^4^ For correlations with ^31^P MRS, ankle dorsiflexion was assessed by manual muscle testing (MMT). In 90% of cases, functional/clinical assessments were performed within 24 h of the MRI exam. The remaining 10% was within a month of the MRI exam. Functional tests were performed following the MRI/^31^P MRS, minimizing any bias induced by muscle strain on the MRI and ^31^P MRS outcomes. The frequency of physical activity reported between ages 10 and 18 years^30^ and CK concentration were also analysed.

### Statistical analysis

Statistical analyses were conducted using SPSS software Version 22 (SPSS, Chicago, IL, USA). The Mann–Whitney tests were performed for comparing demographic data between groups and to assess differences in MRI and ^31^P MRS outcomes between controls and patients, with significance level set at *P* < 0.05.

We also analysed the data using a multilevel linear mixed model (LMM). A first analysis was performed for the MRI parameters to investigate left–right asymmetry, with side as a main variable, adding segment/muscle, group (ambulant vs. non‐ambulant), and site as additional factors, and years since symptom onset and body mass index (BMI), for FF and cCSA, or FF for water T_2_, as continuous predictors to account for these confounding effects. The full analysis also included time point as a main variable and the interaction between time point and group. Random between‐subject variation was also accounted for by including a random intercept in the model, which permitted interpretation of the variance partition coefficient (VPC). For ^31^P MRS, a similar model was used with the predictors being years since symptom onset and FF. The significance level was set at *P* < 0.05. Bonferroni corrections were applied for multiple comparisons for each of the fixed effects.

The Spearman rank correlation test was used to explore additional relationship between MRI and ^31^P MRS variables, and between MRI or ^31^P MRS and functional and strength variables (*P* < 0.05).

The significance level was corrected for comparison of multiple outcome measures (FF, cCSA, and water T_2_ for MRI: *P* = 0.05/3 = 0.017; eight ^31^P MRS indices: *P* = 0.05/8 = 0.006). Additional information on the LMM analysis can be found in the Supporting Information.

For FF and cCSA, standardized response means (SRM) were calculated to assess the sensitivity to change over time (SRM ≥ 0.8 is considered highly sensitive to change).^31^ For water T_2_ and ^31^P MRS indices, both SRM and standardized difference means (SDM)^21^ were assessed.

## Results

### Data overview and demographic differences

**Figure 1:**
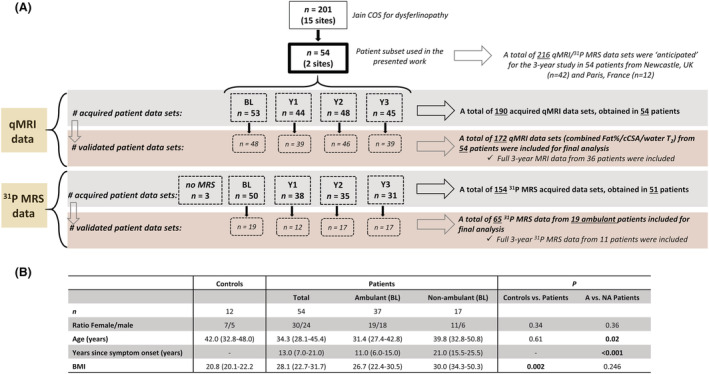
Consort diagram. Missing MRI and ^31^P MRS data sets were due to medical reasons (*x* = 6), equipment failure (*x* = 3), study drop‐out following BL (*x* = 12), or Y2 (*x* = 5). Additionally, ^31^P MRS data were not acquired in those muscles that were highly or completely replaced by fat (*x* = 36), based on visual assessment of the images. Following processing of the image data, 9% of the acquired MRI data were omitted for final analysis due to failed image reconstructions (*x* = 18 of total of 190 data sets). More than half of the ^31^P MRS data were omitted for final analysis due to low SNR, which in most of the cases was due to high FF and/or low residual muscle in the anterior part of the leg. A median FF of 8% corresponded to the ^31^P MRS data that were kept for final analysis, in contrast to the median FF of 43% for those data sets that were omitted. ‘Validated’ means ‘included for final analysis’ following quality control (image series complete, artefact‐free, successful image reconstruction, sufficient signal‐to‐noise ratio). BL, baseline; *n*, number of patients; *x*, number of exams; Y1, Year 1; Y2, Year 2; Y3, Year 3.

*Figure* 1A depicts a flow chart of the data sets acquired in the patients across visits.

*Figure* 1B summarizes the main demographic differences between controls and patients. From the 37 ambulant patients at baseline, 30 were still ambulant at the end of the study. Between LGMD R2/2B and MM patients, and between the two sites, there were no significant differences for age (*P* = 0.56 and *P* = 0.77, respectively), number of years since symptom onset (*P* = 0.64 and *P* = 0.26, respectively), sex (*P* = 0.70 and *P* = 0.66, respectively), BMI, and the ambulant/non‐ambulant patient ratio (*P* = 0.49 and *P =* 0.59, respectively). Only a significant difference in the LGMD R2/MM patient ratio was found between both sites (*P* < 0.001). *Table* S1 summarizes the demographic data for all participants.

### Baseline fat fraction and contractile cross‐sectional area

**Figure 2:**
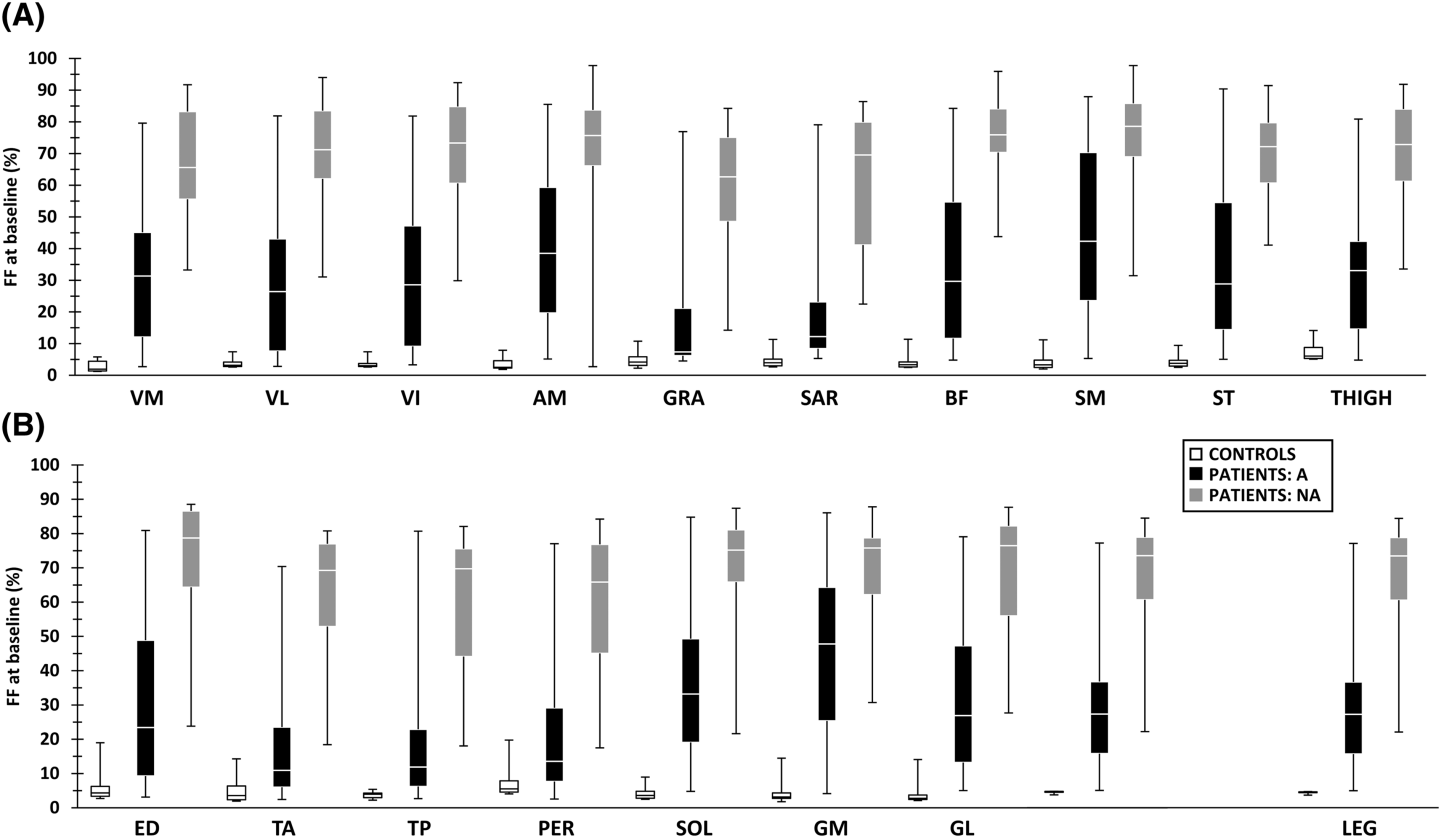
Baseline FF in individual muscles and global segments. *(A)* Thigh baseline FF. *(B)* Leg baseline FF. For all individual muscles and global segments, there were significant differences between controls and ambulant patients, between controls and non‐ambulant patients, and between ambulant and non‐ambulant patients (all *P* < 0.001). More data on baseline FF can be found in *Tables* S2–S4. A, ambulant; AM, adductor magnus; BF, biceps femoris long head; ED, extensor digitorum longus; FF, fat fraction (in %); GL, gastrocnemius lateralis; GM, gastrocnemius medialis; GRA, gracilis; NA, non‐ambulant; PER, peroneus longus; SAR, sartorius; SM, semimembranosus; SOL, soleus; ST, semitendinosus; TA, tibialis anterior; TP, tibialis posterior; VI, vastus intermedius; VL, vastus lateralis; VM, vastus medialis.

**Figure 3:**
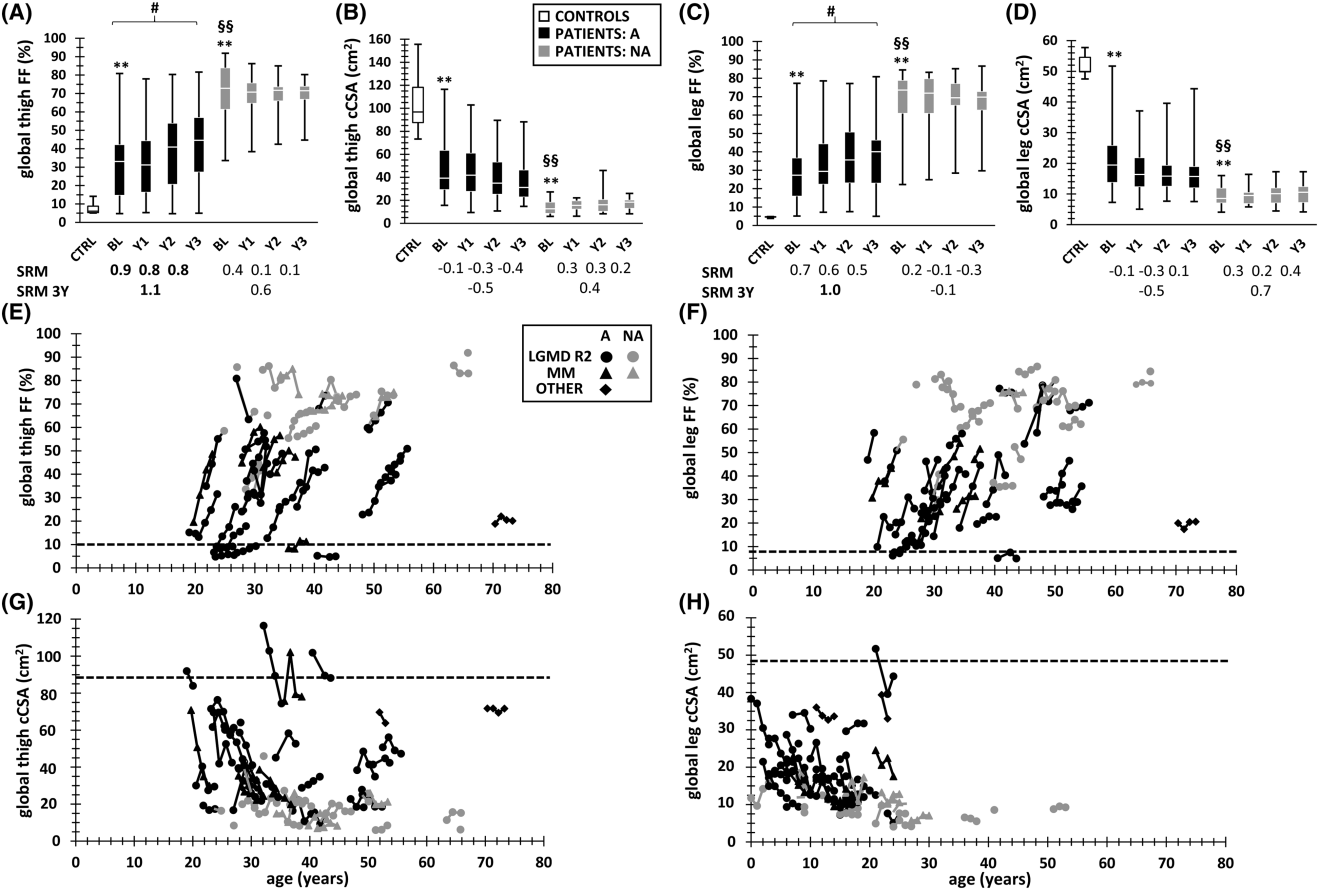
Global FF and cCSA changes over time. *(A)* Global thigh FF. *(B)* Global thigh cCSA. *(C)* Global leg FF. *(D)* Global leg cCSA. *(E)* Global thigh FF trajectories for all individual patients as a function of age. *(F)* Global leg FF trajectories for all individual patients as a function of age. *(G)* Global thigh cCSA trajectories for all individual patients as a function of age. *(H)* Global leg cCSA trajectories for all individual patients as a function of age. Data for both controls, and ambulant and non‐ambulant patients are shown. The symbols in the trajectory plots represent the different phenotypes. The SRM values for annual and three‐year changes in FF and cCSA are depicted beneath the box‐and‐whisker plots *(A)* to *(D)*. The horizontal dashed lines depict the 90th percentile value for FF (thigh: 10.4%, leg: 7.8%) or the 10th percentile value for cCSA (thigh: 87.6 cm^2^, leg: 48.7 cm^2^), as determined in controls. More data can be found in *Tables* S2–S4 for longitudinal FF and cCSA, and in *Table* 1 and *Table* S5 for the LMM analyses. *P < 0.008, **P < 0.001 (between visits); ^#^ *P* < 0.008, ^##^ *P* < 0.001 (between controls and patients); ^§^ *P* < 0.008, ^§§^ *P* < 0.001 (between ambulant and non‐ambulant patients). A, ambulant; BL, baseline; cCSA, contractile cross‐sectional area (in cm^2^); CTRL, controls; FF, fat fraction (in %); LGMD R2, limb‐girdle muscular dystrophy type R2; MM, Miyoshi myopathy; NA, non‐ambulant; SRM, standardized response mean; Y1, Year 1; Y2, Year 2; Y3, Year 3.

Details about significant differences in leg and thigh FF and cCSA between patients and controls can be found in *Figures* 2 and 3A–3D. No significant differences were found between LGMD R2 and MM phenotypes for global thigh (*P* = 0.35) and leg (*P* = 0.71) FF and global thigh (*P* = 0.96) and leg (*P* = 0.37) cCSA.

No significant differences were observed between left and right global FF (global segments: *P* = 0.957, individual muscles: *P* = 0.886) and global cCSA (*P* = 0.035) in patients. All further analyses were performed on the mean of left–right FF and cCSA values.

Highly significant differences were obvious between ambulant and non‐ambulant patients, both for global thigh and leg FF and cCSA (*Table* 1) and for individual muscle FF (*P* < 0.001). While 91% of all ambulant patients demonstrated a global thigh FF ≤ 60% at baseline, 78% of all non‐ambulant patients had a global thigh FF > 60%. Concerning the global segments, expected significant differences were observed for cCSA, but not for FF. Significant differences were evident between individual muscle FF values (*Figure* 2): while thigh and leg posterior muscles showed the highest amount of FF, gracilis, sartorius, tibialis anterior/posterior muscles demonstrated the lowest FF values. No differences between sites were observed for global FF and cCSA and individual FF (*P* = 0.265). There was a significant effect of the number of years since symptom onset on the extent of muscle FF.

**Table 1:** Linear mixed model analysis for global fat fraction, contractile cross‐sectional area, and water T_2_ data

	*β*	SE (*β*)	95% CI	*P*
**Global FF (%)**				
Intercept^a^	26.0	5.5	[15.1 to 37.0]	**<0.001**
Time = Y1	3.2	0.8	[1.6 to 4.8]	**<0.001**
Time = Y2	5.4	0.9	[3.6 to 7.1]	**<0.001**
Time = Y3	7.5	1.1	[5.3 to 9.7]	**<0.001**
Group = NA	28.4	5.8	[16.7 to 40.0]	**<0.001**
Time = Y1*Group = NA	−3.1	1.4	[−5.9 to −0.3]	0.033
Time = Y2*Group = NA	−6.7	1.4	[−9.4 to −4.0]	**<0.001**
Time = Y3*Group = NA	−7.9	1.5	[−10.8 to −5.0]	**<0.001**
Segment = leg	−1.4	1.8	[−5.0 to 2.2]	0.434
Site = Paris	−5.1	5.4	[−15.9 to 5.7]	0.347
Years since onset symptoms	0.7	0.3	[0.1 to 1.2]	**0.017**
BMI	0.01	0.1	[−0.2 to 0.3]	0.904
*(VPC (%)*	*79.4*		*[76.9 to 81.8])*	
**Global cCSA (cm** ^**2**^ **)**				
Intercept^a^	41.8	4.8	[32.3 to 51.4]	**<0.001**
Time = Y1	−1.0	0.8	[−2.5 to 0.6]	0.228
Time = Y2	−2.2	1.0	[−3.9 to −0.6]	**0.009**
Time = Y3	−5.2	1.0	[−7.2 to −3.3]	**<0.001**
Group = NA	−19.6	4.5	[−28.6 to −10.6]	**<0.001**
Time = Y1*Group = NA	2.0	1.5	[−1.0 to 4.9]	0.163
Time = Y2*Group = NA	4.4	1.4	[1.7 to 7.1]	**0.002**
Time = Y3*Group = NA	6.9	1.5	[3.9 to 9.9]	**<0.001**
Segment = leg	−20.0	2.4	[−24.8 to −15.2]	**<0.001**
Site = Paris	2.7	4.0	[−5.4 to 10.8]	0.502
Years since onset symptoms	−26.6	20.8	[−68.3 to 15.1]	0.206
BMI	16.2	12.7	[−8.8 to 41.2]	0.204
*(VPC (%)*	*58.1*		*[55.6 to 60.5])*	
**Global water T**_**2**_ **(ms)**				
Intercept^a^	41.0	0.8	[39.5 to 42.5]	**<0.001**
Time = Y1	0.3	0.4	[−0.5 to 1.0]	0.510
Time = Y2	0.2	0.4	[−0.6 to 0.9]	0.651
Time = Y3	0.3	0.4	[−0.5 to 1.2]	0.461
Group = NA	−1.2	1.0	[−3.3 to 0.8]	0.232
Time = Y1*Group = NA	−1.7	0.8	[−3.2 to −0.2]	0.026
Time = Y2*Group = NA	−3.0	0.7	[−4.5 to −1.6]	**<0.001**
Time = Y3*Group = NA	−3.0	0.8	[−4.6 to −1.4]	**<0.001**
Segment = leg	0.2	0.5	[−0.7 to 1.1]	0.688
Site = Paris	−1.0	0.8	[−2.5 to 0.5]	0.205
Years since onset symptoms	−0.1	0.04	[−0.2 to −0.03]	**0.011**
FF	0.02	0.02	[−0.01 to 0.05]	0.213
*(VPC (%)*	*34.4*		*[28.6 to 40.8])*	

A, ambulant; BMI, body mass index; cCSA, contractile cross‐sectional area; CI, confidence interval; FF, fat fraction; NA, non‐ambulant; SE, standard error; VPC, variance partition coefficient; Y1, Year 1; Y2, Year 2; Y3, Year 3.

Bold values indicate statistically significant differences.

^a^ Estimate for thigh of ambulant patients (group = A) in site = Newcastle at baseline.

More data can be found in *Tables* S2 (for global segment FF and cCSA), S3 and S4 (for individual muscle FF), and S5 (for LMM for individual muscle FF).

### Fat fraction and contractile cross‐sectional area changes over time

**Figure 4:**
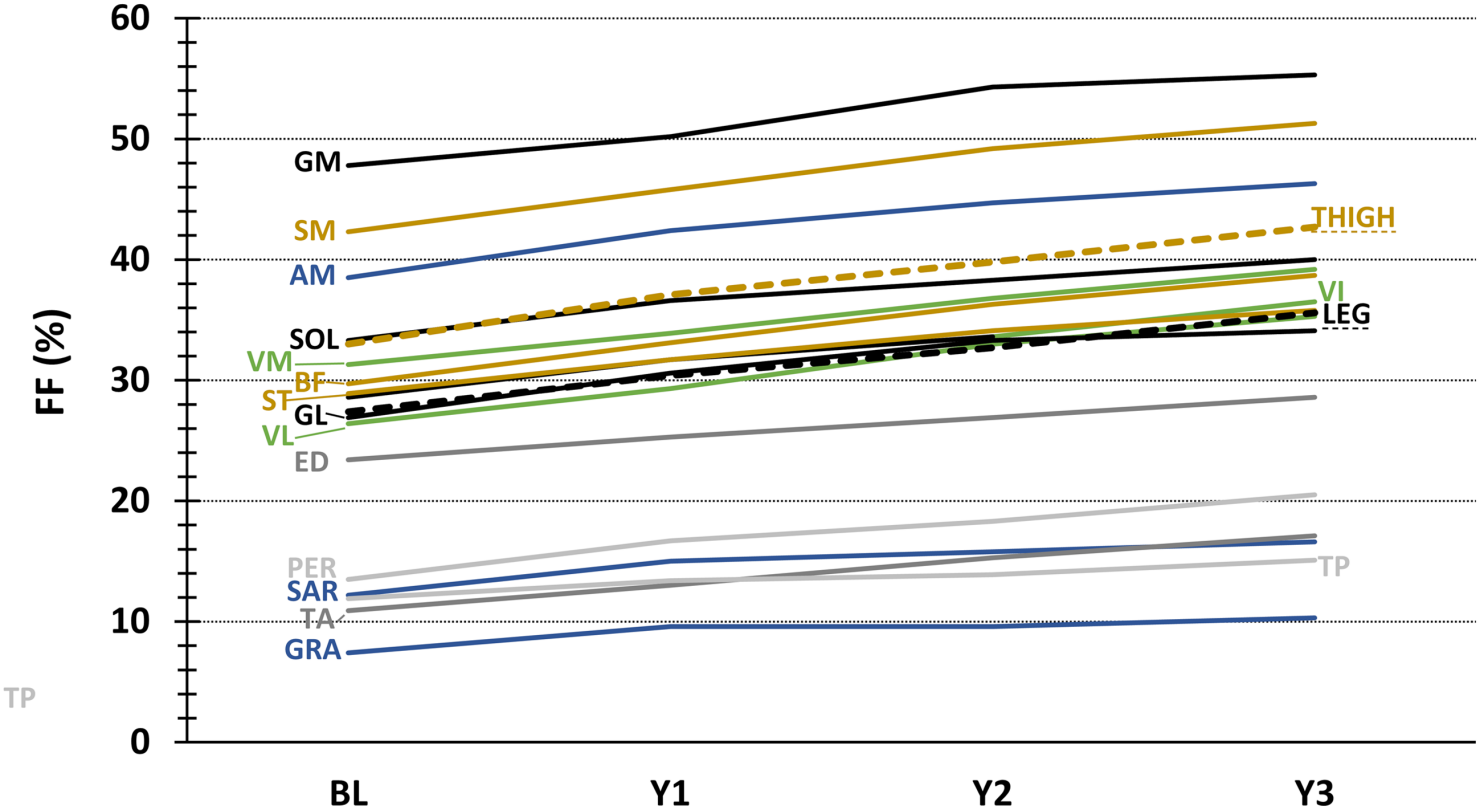
Plot depicting the increase in FF per individual muscle and per segment (thigh and leg) across the 3 years. Every FF value reflects the median in the ambulant study population. AM, adductor magnus; BF, biceps femoris long head; BL, baseline; ED, extensor digitorum longus; FF, fat fraction (in %); GL, gastrocnemius lateralis; GM, gastrocnemius medialis; GRA, gracilis; PER, peroneus longus; SAR, sartorius; SM, semimembranosus; SOL, soleus; ST, semitendinosus; TA, tibialis anterior; TP, tibialis posterior; VI, vastus intermedius; VL, vastus lateralis; VM, vastus medialis; Y1, Year 1; Y2, Year 2; Y3, Year 3.

In ambulant patients, median increases in global FF of 9.6% and 8.4% and median decreases of global cCSA of 11.0% and 12.8% were observed after 3 years, in thigh and leg, respectively (*Figure* 3A–3D). *Figure* 3E–3H illustrates the individual patient trajectories for global FF and cCSA in thigh and leg. Besides the significant changes over time for global FF and cCSA, significant interactions were observed between time and ambulation status, reflecting the differential trajectories between both groups (*Table* 1). The heterogeneity in disease progression between patients is also illustrated by the high values of VPC for both global FF and cCSA. Global thigh FF showed a strong sensitivity to change, with high SRM values for all time points. The analysis of FF in the individual muscles showed the fastest increases in quadriceps, hamstring (biceps femoris long head, semimembranosus), adductor magnus, and posterior leg muscles of ambulant patients (*Figure* 4), with quadriceps muscles showing high SRM values for all time points. Apart from gracilis and gastrocnemius medialis, all other individual muscles in ambulant patients had a 3‐year SRM > 0.8. More data can be found in *Tables* S2 (for global segment FF and cCSA), S3 and S4 (for individual muscle FF), and S5 (for LMM for individual muscle FF).

### Water T_2_

**Figure 5:**
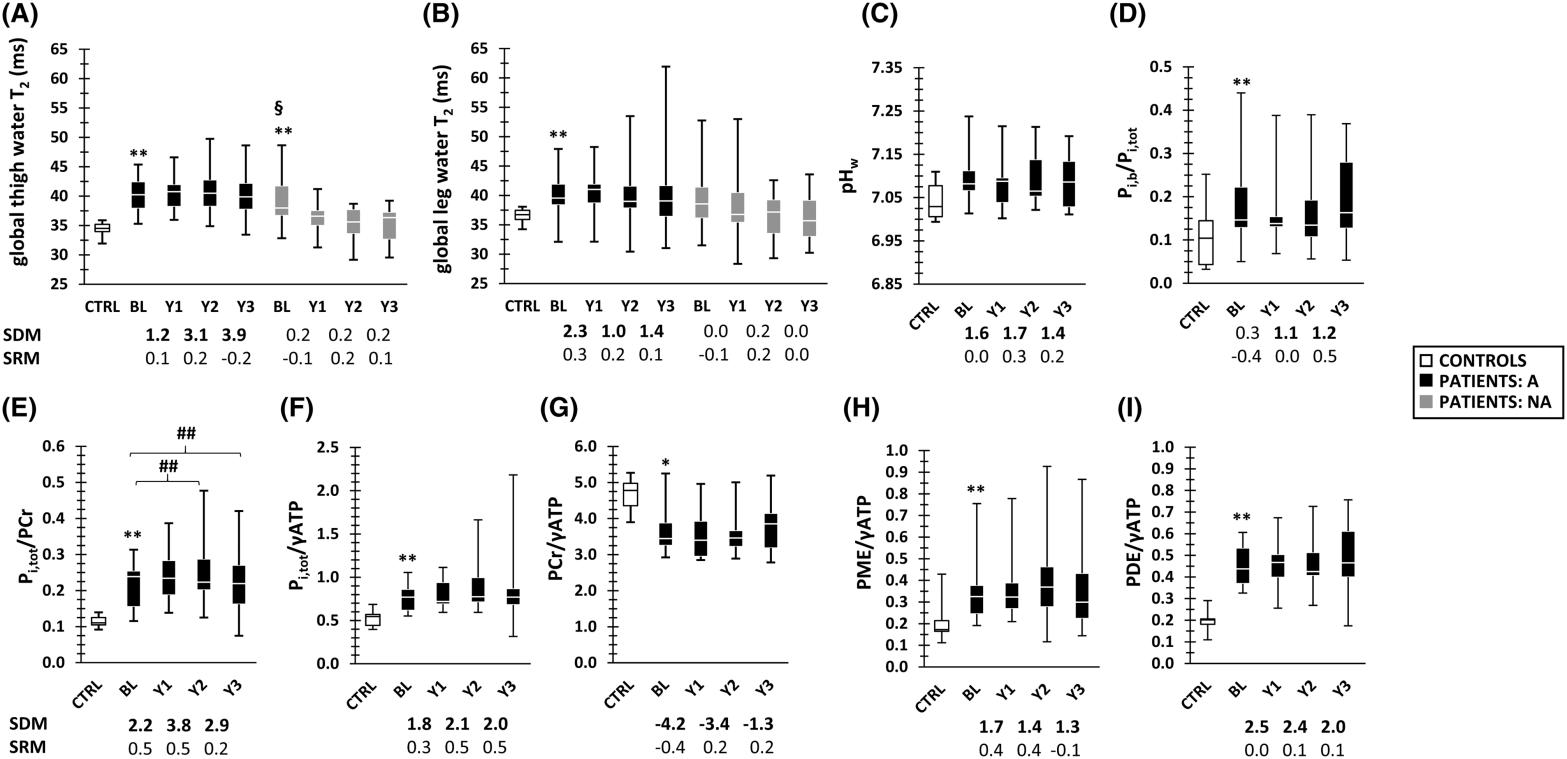
Global water T_2_ and ^31^P MRS changes over time. *(A)* Global thigh water T_2_. *(B)* Global leg water T_2_. *(C)* Anterior leg pH_w_. *(D)* Anterior leg P_i,b_/P_i,tot_. *(E)* Anterior leg P_i,tot_/PCr. *(F)* Anterior leg P_i,tot_/γATP. *(G)* Anterior leg PCr/γATP. *(H)* Anterior leg PDE/γATP. *(I)* Anterior leg PME/γATP. Data for both controls, and ambulant and non‐ambulant patients are shown. The SDM and SRM values for water T_2_ and ^31^P MRS indices are depicted beneath the box‐and‐whisker plots. More data can be found in *Tables* S6–S8 for longitudinal water T_2_ and *Table* S10 for ^31^P MRS, and and *Tables* S9 and S11 for the LMM analyses. *P < 0.008 (water T_2_)/0.006 (^31^P MRS), **P < 0.001 (between visits); ^#^ *P* < 0.008/0.006, ^##^ *P* < 0.001 (between controls and patients); ^§^ *P* < 0.008/0.006, ^§§^ *P* < 0.001 (between ambulant and non‐ambulant patients). A, ambulant; BL, baseline; CTRL, controls; NA, non‐ambulant; PCr, phosphocreatine; PDE, phosphodiesters; pH_w_, weighted pH; P_i,b_, alkaline inorganic phosphate; P_i,tot_, total inorganic phosphate; PME, phosphomonoesters; SDM, standardized difference mean; SRM, standardized response mean; Y1, Year 1; Y2, Year 2; Y3, Year 3; γATP, adenosine diphosphate (γ‐resonance in ^31^P MR spectrum).

Global water T_2_ values were significantly increased in patients as compared with controls (*Figure* 5A and 5B). No significant differences were found between LGMD R2 and MM phenotypes for global thigh (*P* = 0.73) and leg (*P* = 0.72) water T_2_ values.

No significant left–right differences were found in patients for water T_2_ values (global segments: *P* = 0.534, individual muscles: *P* = 0.633). All consequent analyses were performed on the left–right mean water T_2_ values.

No significant differences in water T_2_ were found between ambulant and non‐ambulant patients, both for global (*Figure* 5A‐B, *Table* 1, *Table* S6) and individual muscle values (*P* = 0.796, *Figure* *s* S7‐S8). Unlike global thigh and global leg water T_2_ (*P* = 0.724), significant differences were evident between individual muscle water T_2_ values, with the highest values in the anterior compartments of thigh and leg. The analysis did not demonstrate a significant effect of site on water T_2_. However, when performing an additional analysis on the baseline data only, a difference between both sites became apparent (*P* = 0.006 and *P* = 0.002, for the global and individual muscle values, respectively) but water T_2_ from both sites remained significantly increased as compared with controls. Additionally, FF, as expected, impacted water T_2_, although only observed in individual muscles.

No changes over time in global thigh, global leg, and individual muscle water T_2_ were demonstrated (*Figure* 5A and 5B, *Table* 1, *Table* *s* S6‐S9), but a significant interaction factor of time and group illustrated that differences appeared over time between ambulant and non‐ambulant patients (*Table* 1). The SRM values for water T_2_ were low, but the SDM values were systematically high in ambulant patients.

More data can be found in *Tables* S6 (for global segment water T_2_), S7 and S8 (for individual muscle water T_2_), and S9 (for LMM for individual muscle water T_2_).

### Phosphorus magnetic resonance spectroscopy

Almost all ^31^P MRS indices were significantly abnormal in patients as compared with healthy control values (*Figure* 5C–5I).

No significant effect of site was observed for ^31^P MRS indices (*P*‐values between 0.049 for PCr/γATP and 0.940 for PME/γATP). There were, however, significant effects of FF on pH_w_, P_i,b_/P_i,tot_, PDE/γATP, PME/γATP, and [Mg^2+^] (*P* ≤ 0.005), or of years since symptom onset on P_i,tot_/PCr, PCr/γATP, and P_i,b_/P_i,tot_ (*P* ≤ 0.005).

Except for P_i,tot_/PCr and P_i,tot_/γATP, all other ^31^P MRS indices did not change significantly over time and remained abnormal during the whole study duration (*Figure* 5C–5I). As for water T_2_, the SRM values for ^31^P MRS indices were low, but the SDM values were systematically high.

**Figure 6:**
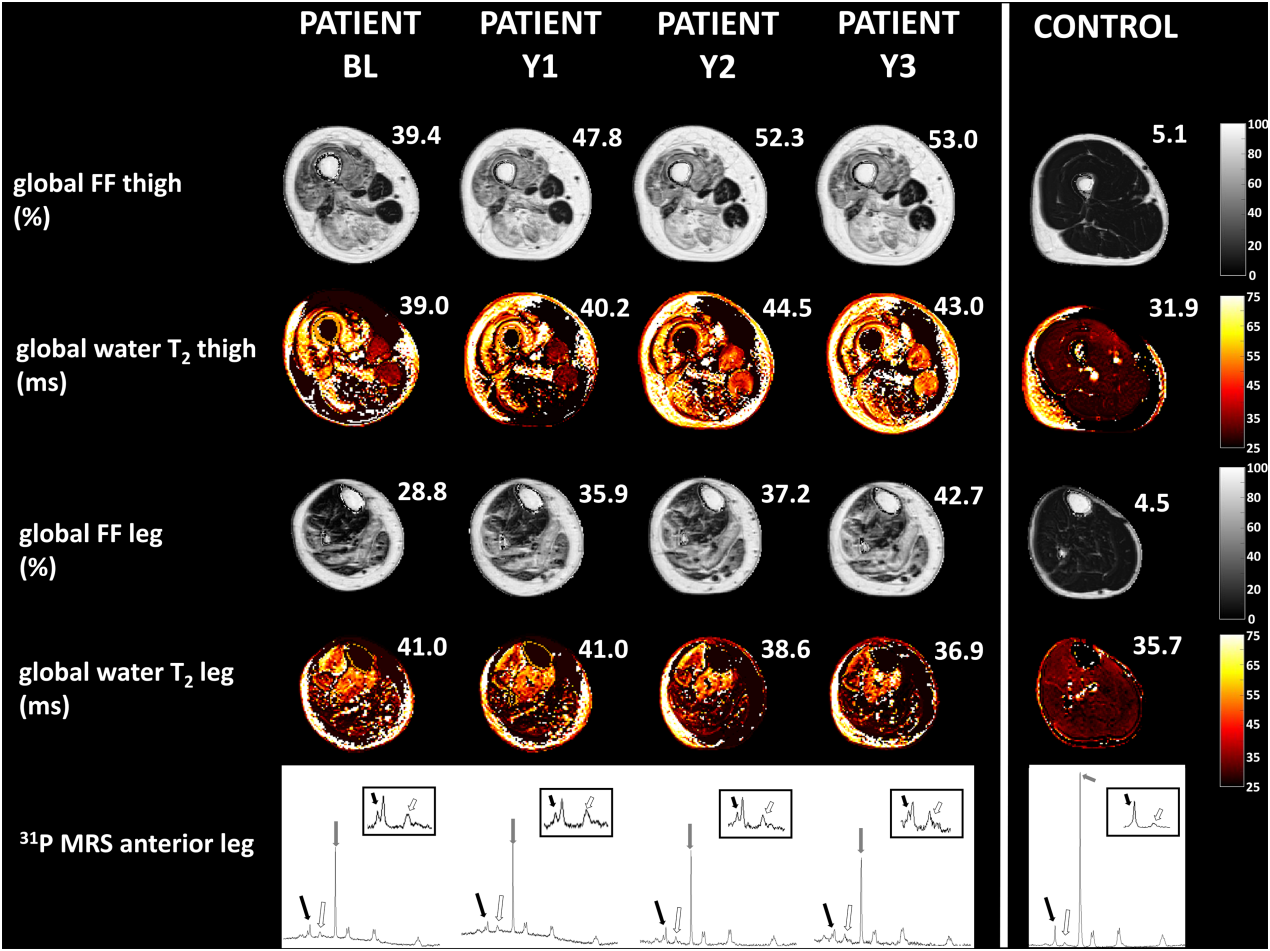
Example of an FF map, a water T_2_ map (for thigh and leg), and a ^31^P MR spectrum (leg anterior part) in a patient across the 3 years (four visits) and in a healthy control subject. Notice the significant increase in FF in both thigh and leg (clearly visible in the anterior part of both segments) and the concomitant elevated water T_2_ values (bright as opposed to the control water T_2_ map). Values for annual global FF and global water T_2_ are also presented. Global FF values exceeding 10.4% (thigh) and 7.8% (leg), and a global water T_2_ exceeding 35.5 ms (thigh) and 37.9 ms (leg) are considered as abnormal (i.e. 90th percentiles determined in control subjects). In the four‐patient ^31^P MR spectra, obtained in the anterior part of the right leg, we notice a clear splitting of the inorganic phosphate resonance (black arrows), an increased PDE resonance (white arrows), and a decreased PCr resonance (grey arrows), which are all quasi‐unchanged across the 3 years, as compared with the healthy control spectrum (where we observe no significant splitting of the inorganic phosphate resonance and a smaller PDE resonance). FF, fat fraction (in %); PCr, phosphocreatine; PDE, phosphodiesters; Y1, Year 1; Y2, Year 2; Y3, Year 3.

*Figure* 6 shows the quantitative FF and water T_2_ maps as well as the ^31^P MR spectra in a patient at all four time points and a healthy volunteer.

More data can be found in *Tables* S10 (for ^31^P MRS indices) and S11 (for LMM for ^31^P MRS indices).

### Relationships between magnetic resonance imaging and spectroscopy variables

**Figure 7:**
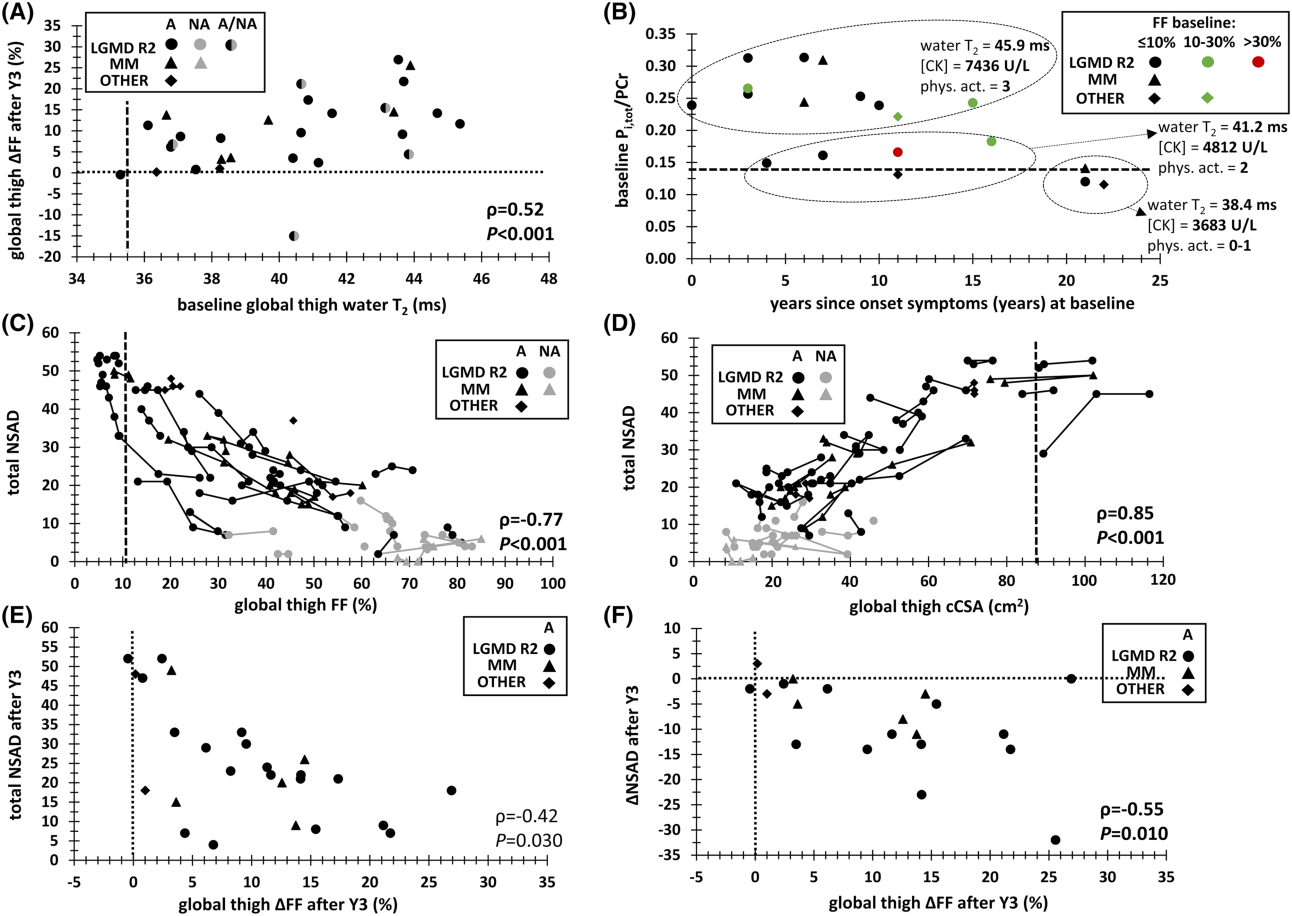
Correlations between MRI, ^31^P MRS, and clinical variables. *(A)* Relationship between baseline global thigh water T_2_ and global change in thigh FF after 3 years: the vertical dashed lines depict the 90th percentile value for global thigh water T_2_ (i.e. 35.5 ms). *(B)* Relationship between years since symptom onset (at baseline) and baseline P_i,tot_/PCr value: the horizontal dashed lines depict the 90th percentile value for anterior leg P_i,tot_/PCr (i.e. 0.13). Investigating mean water T_2_, mean CK values, and the physical activity level during adolescence, three subgroups could be observed. *(C)* Total NSAD trajectories over 3 years for individual patients as a function of global thigh FF: the vertical dashed lines depict the 90th percentile value for global thigh FF (i.e. 10.4%). *(D)* Total NSAD trajectories over 3 years for individual patients as a function of global thigh cCSA: the vertical dashed lines depict the 10th percentile value for global thigh cCSA (i.e. 87.6 cm^2^). *(E)* Relationship between change in global thigh FF and the total NSAD after 3 years (in ambulant patients). *(F)* Relationship between change in global thigh FF and the change in total NSAD after 3 years (in ambulant patients). Spearman rho (*ρ*) correlation values and corresponding *P*‐values are also indicated [the correlation values in figures *(C)* and *(D)* are based on the baseline values]. Physical activity (phys. act.) in teenage years has been investigated in Jain COS patients in a publication by Moore *et al*.,^30^ with 0 = reported no physical activity, 1 = reported vigorous activity occasionally/monthly or moderate activity weekly, 2 = reported moderate activity multiple times a week or vigorous activity once weekly, 3 = reported vigorous activity multiple times a week. [CK], creatine kinase concentration (in U/L); A, ambulant; cCSA, contractile cross‐sectional area (in cm^2^); FF, fat fraction (in %); LGMD R2, limb‐girdle muscular dystrophy type R2; MM, Miyoshi myopathy; NA, non‐ambulant; NSAD, North Star Assessment for limb‐girdle muscular Dystrophies; PCr, phosphocreatine; phys. ΔFF, change in FF (in %, absolute); P_i,tot_, total inorganic phosphate; ΔNSAD, change in total NSAD value.

Global thigh water T_2_ was significantly correlated to ΔFF after 3 years (*ρ* = 0.52, *P* < 0.001), although less for ΔcCSA (*ρ* = −0.37, *P* = 0.002) (*Figure* 7A). In individual muscles, significant correlations between mean water T_2_ and ΔFF were observed for the vasti muscles and biceps femoris long head, gracilis, soleus, and extensor digitorum muscles (*ρ* = 0.50–0.60, *P* < 0.001). We found that global thigh water T_2_ was correlated with the CK concentration (*ρ* = 0.48, *P* = 0.002). Water T_2_ values were also significantly correlated with ^31^P MRS indices such as P_i,tot_/PCr (*ρ* = 0.69, *P* = 0.001) and PCr/γATP (*ρ* = −0.63, *P* = 0.004). From an exploratory perspective, we illustrated in *Figure* 7B the relationship between P_i,tot_/PCr, time since onset of symptoms, water T_2_, CK levels, and physical activity regime in adolescence. In *Tables* S12 and S13, the correlation analyses are summarized.

### Relationships between magnetic resonance imaging/spectroscopy and functional strength variables

Muscle function as measured by the NSAD decreased steadily with increasing FF and decreasing cCSA (*Figure* 7C and 7D). A significant relationship existed between the change in total NSAD score and FF after 3 years in ambulant patients (*Figure* 7E and 7F).

## Discussion

The presented 3‐year quantitative MRI and ^31^P MRS study has demonstrated cumulative muscle degradation in legs and thighs, as measured by the yearly and overall increases in FF and decreases in cCSA. Furthermore, muscle water T_2_ values and various ^31^P MRS indices have proven to be highly and persistently abnormal and were correlated to disease progression as evaluated by an increase in FF.

### Study demographics, extent of disease, and disease progression

The investigated study cohort adequately represented the population in the full Jain COS for dysferlinopathy, as described in the T_1_‐weighted MRI study.^8^ As expected, in line with earlier publications,^5^, ^6^, ^7^, ^8^, ^32^ no differences were shown between LGMD R2 and MM phenotypes. The quantitative MRI findings revealed a strong proximo‐distal and posterior dominant pattern of muscle fat replacement in 80% of the examined patients, which is slightly less than the 88% reported in Angelini *et al*.^33^ but much higher than the 56% in Jin *et al*.^7^ Upper limb, shoulder, trunk, and pelvis muscles (not investigated in the current study) are also known to be affected,^6^, ^8^, ^9^ but it is known that this follows the early posterior thigh and leg compartment involvement. Muscle fat replacement was heterogeneous across the patient population, and the pronounced involvement of medio‐posterior muscles such as semimembranosus, adductor magnus, and gastrocnemius medialis, as compared with anterior muscles, with an absence of a significant left–right asymmetry confirm the results of previous studies using T_1_‐weighted MRI.^5^, ^6^, ^7^, ^8^, ^9^, ^33^ Muscle involvement in dysferlinopathy is similar to observations in other forms of LGMD. In LGMD R1/2A^34^ and LGMD R9/2I,^19^ however, anterior muscles are less impacted in LGMD R9/2I, whereas, in LGMD R12/2L,^35^ a higher level of asymmetry might be present.

The annual increase in global FF of 3–4%, and concomitant decrease in cCSA, as found in both thighs and legs of ambulant patients, reflect a considerably faster rate of disease progression compared with the ≈1% increase in muscle fat replacement found after 1 year in LGMD R9 (formerly LGMD2I).^19^ Despite the significant annual disease progression in dysferlinopathy, there is a large inter‐individual variability, which is more pronounced than in LGMD R9.^24^ Nevertheless, the knowledge of the annual expected FF increase will enable us to establish clinically meaningful outcome measures for future therapeutic trials. Using global values for FF is a valid approach for evaluating disease progression compared with individual ROIs, as demonstrated by the high SRM values.^17^, ^19^, ^23^, ^24^

### Disease activity

Skeletal muscle water T_2_ is another quantitative MRI outcome measure, one that is non‐specific, yet sensitive to mechanisms such as inflammation and oedema, and reflects the disease activity.^11^ Water T_2_ was, in general, significantly elevated in patients, and the highest values were found in the anterior parts of thigh and leg, which were, on average, less replaced by adipose tissue. This confirms findings of earlier studies where hyperintensities found on T_2_‐weighted images and classified as oedema preceded muscle fat replacement in muscles that were in the early stages of the disease.^5^, ^6^, ^7^ Indeed, we found that patients with higher water T_2_ demonstrated faster disease progression as muscle fat replacement increases were more pronounced, a relationship that was established in other myopathies with both a known inflammatory component such as dermatomyositis,^36^ inclusion body myositis,^20^ FSHD,^18^ and DMD,^13^, ^14^ but also in late‐onset Pompe disease^22^ and GNE myopathy.^21^ In muscles such as gracilis and sartorius, where FF was low and disease progression was slow, water T_2_ was found to be in normal ranges. In a study in DMD, it was demonstrated that water T_2_ was significantly increased even before any signs of muscle fat replacement.^14^ In the current study, the water T_2_ was found to be systematically increased in both thigh and leg of patients over the course of the entire study, especially in ambulant patients, indicating that the disease activity mechanisms are persistent throughout the disease duration and might endure as long as 18 years, as reported earlier.^6^

The different ^31^P MRS indices were also found to be continuously abnormal over the 3 years. This included a high level of PDE/γATP, associated with an increased phospholipid turnover, and an elevated alkaline pH_w_, reflecting an increased intracellular pH or increased interstitial P_i,tot_ pool,^13^ both indices pointing towards a disturbance at the level of the sarcolemma, and found earlier in DMD,^13^, ^14^ Becker muscular dystrophy,^16^ FSHD,^18^ and GNE myopathy.^21^ The ^31^P MRS data were generally obtained in patients with very low muscle fat replacement, revealing that changes in disease activity can be measured before the occurrence of any macroscopic muscle destruction, as demonstrated in DMD.^14^ We also found that the shorter the disease duration, the higher the level of P_i,tot_/PCr was, which is an index that reflects the degree of muscle energy wasting. Interestingly, the patients with the highest P_i,tot_/PCr also seemed to have a history of a more vigorous physical activity regime,^30^ as compared with patients with lower P_i,tot_/PCr, which might be an indication that there is a potential negative effect of physical exercise in dysferlinopathy patients.^33^ As exercise is usually found to be beneficial in neuromuscular disorders,^37^ this might also have implications for future treatment of these patients, including physical therapy.

The persistent abnormal values for water T_2_ and most ^31^P MRS indices in dysferlinopathy render their corresponding SRM values indicative of a low sensitivity to change. However, the fact that these MRI and ^31^P MRS variables are all significantly different compared with normal muscle (i.e. high SDM^21^) makes them very useful biomarkers for assessing the impact of a potential therapeutic intervention, especially in the early stages of the disease.

### Relationship with function and strength

Similar to the earlier larger‐scale dysferlinopathy studies,^5^, ^6^, ^7^, ^9^ a strong relationship was observed between muscle fat replacement, disease duration, and function. However, the Jain COS study was the first study where an LGMD‐specific scale of motor performance was used.^4^ The strong correlation found between total NSAD and global thigh FF, especially in the earlier stages of the disease, as measured with FF values between 0% and 40%, is important in the search for clinically meaningful outcome measures. In contrast to the 6 min walk distance where a sudden and very strong decline was observed only after FF exceeded 60%,^15^ the NSAD is highly sensitive to changes in FF in the earlier disease progression. When the investigated endpoint is the loss of ambulation, the complementarity of the concomitant investigations of total NSAD and quantitative MRI proves its worth, especially in light of potential treatments.

### Methodological issues

First, the major methodological issue is the multi‐centre aspect of this study. The use of different MRI clinical systems introduced a certain degree of variability, although the differences were found to be negligible between Newcastle and Paris, at least in healthy controls. In patients, however, we observed significant differences in water T_2_ between sites, but at both sites, values were significantly abnormal as compared with control values. This issue underlines, besides the obvious necessity to harmonize MRI protocols between sites in multi‐centre studies, the fact that water T_2_ results always need to be interpreted in their proper context. Moreover, as a quantitative MRI outcome measure for disease activity, water T_2_, with its potential to predict a subsequent change in muscle fat replacement, is obviously more relevant in the early stages of the disease. Second, water T_2_ values were determined using the tri‐exponential fitting procedure.^25^ Similarly, as observed in previous studies reporting on water T_2_ in fatty replaced muscle,^25^, ^38^, ^39^, ^40^ the water T_2_ derived from the tri‐exponential fitting has been shown to be abnormally low at high FF values. Although this result agrees with what has been observed when using the ‘gold standard’ MRS‐based method,^40^ further research is still needed to explain this observation. More recently, water T_2_ mapping procedures based on the extended phase graph (EPG) algorithm, which avoid the need to acquire an additional B_1_^+^ map, have demonstrated their use in fatty replaced muscle.^38^, ^39^ Although these EPG methods are promising, in the present work, we have chosen to use the tri‐exponential fitting procedure because, besides being a validated method, it is the technique employed in most of our earlier published works involving clinical applications (some of them cited in the current work^13^, ^21^, ^22^), thus allowing for a coherent comparison between these studies.

## Conclusions

This study provided quantitative natural history MRI and ^31^P MRS data in a moderately large group of dysferlinopathy patients with a heterogeneous disease state. Although challenging when organized in a multi‐centre set‐up, the investigated quantitative outcome measures were shown to be sensitive to annual changes (in case of FF) or were persistently abnormal and correlated to subsequent changes in FF (in case of water T_2_ and ^31^P MRS). Evaluating a global segment instead of all individual muscles separately has not only obvious advantages in processing and analysis but also proved to be as valuable and as sensitive (as individual muscles) in evaluating disease progression and activity in dysferlinopathy. Quantitative MRI/S outcomes, identifying changes in muscle structure, pathophysiology, and metabolism, were shown to change over time. Adding functional tests, such as the total NSAD score, can link these changes to clinically meaningful endpoints. The quantitative data acquired in this study can be used as reference values for future clinical trials in dysferlinopathy and to compare with similar quantitative MRI/S data in other LGMD subtypes. New longitudinal quantitative MRI/^31^P MRS studies might be anticipated in other anatomical targets, such as trunk, pelvis, and/or upper limb muscles, as we know they are affected further downstream in the disease progression of dysferlinopathy. At the time of publication, the Jain COS2 study is ongoing, which includes quantitative MRI in lower and upper limb.

## Funding

The Jain Foundation has provided an estimated $4 million USD to fund this study.

## Conflicts of interest

A.M.B., A.M., B.M., E.C.A.A., F.E.S., H.R., I.W., J.M.B., J.L.L., K.B., L.R., M.J., M.K.J., N.A., P.G.C., U.M., and V.S. report grants from JAIN Foundation, during the conduct of the study. A.C., D.W., J.Y.H., J.D.M., L.W., R.F.T., T.S., and T.H. report no competing interests.

## Supporting information




**Table S1.** Demographics of the overall patient cohort (54 patient from the Jain Foundation COS).
**Table S2.** Global segment baseline FF and cCSA values in controls and patients, and annual and 3‐year ΔFF and ΔcCSA values in patients, in thigh and leg.
**Table S3.** Individual thigh muscle baseline FF values in controls and patients, and annual and 3‐year ΔFF values in patients.
**Table S4.** Individual leg muscle baseline FF values in controls and patients, and annual and 3‐year ΔFF values in patients.
**Table S5.** Linear Mixed Model analysis for individual muscle water FF.
**Table S6.** Global segment baseline water T_2_ values in controls and patients, and annual and 3‐year average water T_2_ values in patients, in thigh and leg.
**Table S7.** Individual thigh muscle baseline water T_2_ values in controls and patients, and annual and 3‐year average water T_2_ values in patients.
**Table S8.** Individual leg muscle baseline water T_2_ values in controls and patients, and annual and 3‐year average water T_2_ values in patients.
**Table S9.** Linear Mixed Model analysis for individual muscle water T2.
**Table S10.** Baseline ^31^P MRS values in controls and patients, and annual and 3‐year average ^31^P MRS values values in patients, in the anterior leg compartment.
**Table S11.** Linear Mixed Model analysis for ^31^P MRS data.
**Table S12.** Correlation analysis of global segment quantitative MRI indices, functional and clinical parameters.
**Table S13.** Correlation analysis of ^31^P MRS indices and quantitative MRI in anterior leg compartment and functional and clinical parameters.
