# Supplementary material for: Three‐year quantitative magnetic resonance imaging and phosphorus magnetic resonance spectroscopy study in lower limb muscle in dysferlinopathy
Source: J Cachexia Sarcopenia Muscle. 2022 Apr 3;13(3):1850–63. doi: 10.1002/jcsm.12987 (PMC9178361; doi:10.1002/jcsm.12987)
Supplement: Supplementary file 1 — Table S1. Demographics of the overall patient cohort (54 patient from the Jain Foundation COS). Table S2. Global segment baseline FF and cCSA values in controls and patients, and annual and 3‐year ΔFF and ΔcCSA values in patients, in thigh and leg. Table S3. Individual thigh muscle baseline FF values in controls and patients, and annual and 3‐year ΔFF values in patients. Table S4. Individual leg muscle baseline FF values in controls and patients, and annual and 3‐year ΔFF values in patients. Table S5. Linear Mixed Model analysis for individual muscle water FF. Table S6. Global segment baseline water T2 values in controls and patients, and annual and 3‐year average water T2 values in patients, in thigh and leg. Table S7. Individual thigh muscle baseline water T2 values in controls and patients, and annual and 3‐year average water T2 values in patients. Table S8. Individual leg muscle baseline water T2 values in controls and patients, and annual and 3‐year average water T2 values in patients. Table S9. Linear Mixed Model analysis for individual muscle water T2. Table S10. Baseline 31P MRS values in controls and patients, and annual and 3‐year average 31P MRS values values in patients, in the anterior leg compartment. Table S11. Linear Mixed Model analysis for 31P MRS data. Table S12. Correlation analysis of global segment quantitative MRI indices, functional and clinical parameters. Table S13. Correlation analysis of 31P MRS indices and quantitative MRI in anterior leg compartment and functional and clinical parameters. [file JCSM-13-1850-s001.docx]

**SUPPORTING INFORMATION**

**Title: Three-year quantitative MRI and ^31^P MRS study in lower limb muscle in dysferlinopathy**

**Authors:** Harmen Reyngoudt, Fiona E. Smith, Ericky C.A. Araújo, Ian Wilson, Roberto Fernández Torrón, Meredith K. James, Ursula R. Moore, Jordi Díaz-Manera, Benjamin Marty, Noura Azzabou, Heather Gordish, Laura Rufibach, Tim Hodgson, Dorothy Wallace, Louise Ward, Jean-Marc Boisserie, Julien Le Louër, Heather Hilsden, Helen Sutherland, Aurélie Canal, Jean-Yves Hogrel, Marni Jacobs, Tanya Stojkovic, Kate Bushby, Anna Mayhew, Jain Clinical Outcome Study for Dysferlinopathy consortium, Volker Straub, Pierre G. Carlier, Andrew M. Blamire

**Corresponding author:** Andrew M. Blamire

Affiliation: Magnetic Resonance Centre, Translational and Clinical Research Institute, Faculty of Medical Sciences, Newcastle University, Newcastle upon Tyne, UK

E-mail address: Andrew.Blamire@newcastle.ac.uk

**Supplementary Text: Statistical analysis**

Statistical analyses were conducted using SPSS software version 22 (SPSS, Chicago, IL, USA).

**STEP 1:**

1. Mann-Whitney tests were used to comparing demograpic variables between controls and patients and between the different patient groups (ambulant vs. non-ambulant patients, LGMD R2 vs. Miyoshi myopathy patients, Newcastle-included vs. Paris-included patients):

- for controls: sex and age

- for patients: sex, age, years since onset symptoms, BMI, ratio of ambulant/non-ambulant

2. Mann-Whitney tests were also used to compare the different qMRI and ^31^P MRS outcomes between controls and patients.

**STEP 2:**

1. As the full data set includes multiple within-subject values (muscles, left-right, time points), we opted to use a linear mixed model (LMM).

2. Based on the results of the initial statistical tests (STEP 1) and some additonal correlation analyses (to assess the relationship between some of the investigated parameters), a hypothesis-driven LMM was applied to the full patient data set. For instance, based on preliminary analyses, sex and phenotype were not added to the model (both factors did not contribute to explain differences).

The LMM model included (for FF, cCSA, water T_2_):

- time (as main within-subject factor)

- segment or muscle (as an additional within-subject factor)

*- side (left vs. right, as an additional within-subject factor)*

- group (ambulant vs non-ambulant, as a between-subject factor)

- time*group interaction factor

- investigator site (as an an additional between-subject factor)

- years since onset symptoms (as a continous covariate)

- BMI (as a continous covariate) 🡺 only for FF, cCSA

- FF (as a continous covariate) 🡺 only for water T2

Additional:

- Autoregressive first order covariance structures were used for the repeated measures.

- Random between-subject variation was also accounted for by, including a random intercept in the model. A scaled identity covariance structure was used.

3. An intitial LMM was performed on the baseline data (so without time within-subject factor) to investigate the left-right asymmetry in patients. As this was systematically not significant (for FF, cCSA and water T2) for both global segments and individual muscle analysis, side was eliminated for the final LMM analyses.

4. As it was a hypthesis-driven approach, the LMM models used were slightly different between FF/cCSA and water T2, as can be understood from STEP2.2. Nevertheless, the core structure of the model was kept similar for all parameters and was only modified for the confounding covariates (BMI, FF).

As such, the LMM analyses for the 31P MRS indices was also different, as only data from ambulant patients from a single muscle group were analyzed. The LMM analysis included:

- time (as main within-subject factor)

- investigator site (as an an additional between-subject factor)

- years since onset symptoms (as a continous covariate)

- FF (as a continous covariate)

Additional:

- Autoregressive first order covariance structures were used for the repeated measures.

- No random intercept was added to this model.

- Values for beta, beta standard error, the 95% confidence interval, and *P-value* are given for all predictors. The estimated proportion of variance due to between-subject variability is presented by the variance partition coefficient (VPC).

**Supplementary Table 1 Demographics of the overall patient cohort (54 patient from the Jain Foundation COS).**

| **Patient n°** | **Site** | **Phenotype at onset** | **Sex** | **BMI ^a^** | **Age at BL (years)** | **Disease duration at BL (years)** | **Ambul-atory status at BL** | **Total NSAD at BL^b^** | **Physical activity in teenage years ^c^** | **CK at BL^b^ (U/L)** |
| --- | --- | --- | --- | --- | --- | --- | --- | --- | --- | --- |
| 1 | Newcastle | LGMD R2 | M | n/a | 28.2 | 3 | A | n/a | 3 | 9531 |
| 2 | Newcastle | LGMD R2 | F | 37.9 | 46.9 | 23 | A | 18 | 2 | 2916 |
| 3 | Newcastle | LGMD R2 | M | 24.9 | 43.3 | 21 | N | n/a | 3 | 1696 |
| 4 | Newcastle | LGMD R2 | F | 22.1 | 34.2 | 7 | A | 44 | 3 | 2919 |
| 5 | Newcastle | LGMD R2 | F | 42.2 | 40.8 | 15 | A | 5 | 0 | 1194 |
| 6 | Newcastle | LGMD R2 | M | 24.9 | 36.2 | 15 | N | n/a | 2 | 5594 |
| 7 | Newcastle | LGMD R2 | M | 37.6 | 26.9 | 11 | A | 5 | 3 | 4024 |
| 8 | Newcastle | LGMD R2 | F | 38.2 | 31.3 | 17 | N | n/a | 1 | 3056 |
| 9 | Newcastle | LGMD R2 | F | 29.4 | 23.6 | 13 | A | 33 | 2 | 13134 |
| 10 | Newcastle | LGMD R2 | M | 17.4 | 20.6 | 4 | A | 21 | 2 | 20662 |
| 11 | Newcastle | LGMD R2 | M | 33.5 | 28.5 | 11 | N | n/a | 2 | 13380 |
| 12 | Newcastle | LGMD R2 | F | 32.5 | 27.0 | 9 | N | n/a | 3 | 5657 |
| 13 | Newcastle | LGMD R2 | M | 22.0 | 37.1 | 13 | A | 18 | 3 | n/a |
| 14 | Newcastle | LGMD R2 | F | 21.7 | 44.0 | 24 | N | n/a | 3 | 842 |
| 15 | Newcastle | LGMD R2 | F | 24.4 | 38.6 | 15 | A | n/a | 2 | 4270 |
| 16 | Newcastle | LGMD R2 | F | 24.2 | 49.2 | 10 | A | 24 | 0 | 1961 |
| 17 | Newcastle | LGMD R2 | M | 23.9 | 48.1 | 16 | A | 34 | 2 | 1755 |
| 18 | Newcastle | LGMD R2 | M | 28.9 | 52.5 | 18 | A | n/a | 0 | 2273 |
| 19 | Newcastle | LGMD R2 | M | 23.9 | 27.8 | 6 | A | 13 | 2 | n/a |
| 20 | Newcastle | LGMD R2 | M | 31.5 | 47.0 | 15 | A | n/a | 0 | 2492 |
| 21 | Newcastle | Miyoshi | M | 30.3 | 34.3 | 16 | N | n/a | 2 | 5147 |
| 22 | Newcastle | LGMD R2 | F | 28.1 | 32.6 | 15 | A | 16 | 2 | 4782 |
| 23 | Newcastle | LGMD R2 | F | 29.2 | 50.8 | 16 | A | 31 | 0 | 2996 |
| 24 | Newcastle | LGMD R2 | F | 38.0 | 44.9 | 8 | A | 14 | 0 | 3162 |
| 25 | Newcastle | Miyoshi | F | 50.3 | 30.1 | 0 | N | n/a | 2 | 3286 |
| 26 | Newcastle | LGMD R2 | F | 23.2 | 23.1 | 0 | A | 53 | n/a | 4810 |
| 27 | Newcastle | LGMD R2 | F | 33.2 | 65.8 | 41 | N | n/a | 0 | 891 |
| 28 | Newcastle | Miyoshi | F | 30.8 | 50.3 | 22 | N | n/a | 2 | n/a |
| 29 | Newcastle | LGMD R2 | M | 25.0 | 29.9 | 9 | A | 24 | 3 | 7704 |
| 30 | Newcastle | LGMD R2 | F | 47.8 | 60.2 | 24 | N | n/a | 3 | 1065 |
| 31 | Newcastle | LGMD R2 | M | 25.7 | 37.2 | 21 | N | n/a | 3 | 2140 |
| 32 | Newcastle | LGMD R2 | F | 26.0 | 35.6 | 18 | N | n/a | 0 | 1233 |
| 33 | Newcastle | LGMD R2 | M | 31.9 | 32.1 | 3 | A | 45 | 3 | 11352 |
| 34 | Newcastle | LGMD R2 | F | 17.1 | 28.6 | 11 | A | 28 | 2 | n/a |
| 35 | Newcastle | Miyoshi | M | n/a | 27.9 | 12 | A | n/a | 3 | 3323 |
| 36 | Newcastle | Miyoshi | M | 21.8 | 29.0 | 11 | A | 29 | 2 | n/a |
| 37 | Newcastle | LGMD R2 | F | 30.5 | 18.0 | 1 | A | 49 | 0 | 4533 |
| 38 | Newcastle | Miyoshi | F | 22.6 | 35.7 | 15 | A | 49 | 3 | 2496 |
| 39 | Newcastle | LGMD R2 | F | 30.0 | 63.4 | 51 | N | n/a | 3 | 1031 |
| 40 | Newcastle | LGMD R2 | F | 22.4 | 27.0 | 3 | A | 46 | 2 | n/a |
| 41 | Newcastle | LGMD R2 | M | 35.6 | 25.4 | 6 | A | 44 | 2 | 12932 |
| 42 | Newcastle | LGMD R2 | F | 21.0 | 23.4 | 4 | A | 51 | 3 | n/a |
| 43 | Paris | Miyoshi | F | 19.3 | 41.3 | 27 | N | n/a | 3 | 1696 |
| 44 | Paris | Miyoshi | F | 28.5 | 19.7 | 7 | A | 32 | 2 | 11429 |
| 45 | Paris | other | M | 22.8 | 70.3 | 11 | A | 45 | 1 | 2671 |
| 46 | Paris | LGMD R2 | M | 26.7 | 40.5 | 21 | A | 54 | n/a | 2097 |
| 47 | Paris | LGMD R2 | F | 20.1 | 51.3 | 36 | N | n/a | 3 | 940 |
| 48 | Paris | other | F | 29.4 | 51.2 | 22 | A | 36 | 2 | 4741 |
| 49 | Paris | other | F | 34.5 | 28.4 | 11 | A | 21 | 3 | 3551 |
| 50 | Paris | Miyoshi | M | 19.4 | 33.7 | 13 | A | 20 | 3 | 7239 |
| 51 | Paris | Miyoshi | M | 23.1 | 39.8 | 22 | N | n/a | 3 | 1168 |
| 52 | Paris | Miyoshi | M | 28.4 | 27.8 | 21 | A | 28 | 1 | 4285 |
| 53 | Paris | Miyoshi | F | 27.3 | 31.4 | 6 | A | 20 | 2 | 3232 |
| 54 | Paris | LGMD R2 | M | 20.4 | 21.8 | 6 | A | 20 | 3 | 6126 |

A = ambulant, BL = baseline, BMI = body-mass index, CK = creatine kinase concentration (in units per liter), LGMD R2 = limb-girle muscle dystrophy type R2, Miyoshi = Miyoshi myopathy, NA = non-ambulant, n/a = not available, NSAD = North Star Assessment for limb-girdle muscular Dystrophies, other = proximo-distal form of dysferlinopathy or non-defined

^a^ For these two patients, this information was not available. Both patients dropped out of the study after the baseline visit.

^b^ For these patients, this information was not available at baseline.

^c^ Physical activity in teenage years has been investigated in Jain COS patients in a publication by Moore et al. [30] (0 = reported no physical activity, 1 = reported vigorous activity occasionally/monthly or moderate activity weekly, 2 = reported moderate activity multiple times a week or vigorous activity once weekly, 3 = reported vigorous activity multiple times a week). For two patients, this information was not available.

**Supplementary Table 2 Global segment baseline FF and cCSA values in controls and patients, and annual and 3-year ΔFF and ΔcCSA values in patients, in thigh and leg**

| **segment** | **group** | **FF at BL** | | **ΔFF between BL and Y1** | | | **ΔFF between Y1 and Y2** | | | **ΔFF between Y2 and Y3** | | | **ΔFF after 3 years** | | |
| --- | --- | --- | --- | --- | --- | --- | --- | --- | --- | --- | --- | --- | --- | --- | --- |
|  |  | ***n*** | **Med (IQD)** | ***n*** | **Med (IQD)** | **SRM** | ***n*** | **Med (IQD)** | **SRM** | ***n*** | **Med (IQD)** | **SRM** | ***n*** | **Med (IQD)** | **SRM** |
| THIGH | controls | 12 | 6.1 (5.3-8.8) |  | | |  | | |  | | |  | | |
|  | patients: A | 34 | 33.0 (14.6-42.3)**^**^** | 26 | 4.1 (1.3-6.2) | **0.9** | 26 | 2.7 (1.0-6.7) | **0.8** | 30 | 2.9 (0.8-5.8) | **0.8** | 30 | 9.6 (3.5-14.8) | **1.1** |
|  | patients: NA | 14 | 72.8 (61.2-84.0)**^**^** | 12 | 0.8 (-0.6-4.3) | 0.4 | 12 | 0.4 (-0.4-2.8) | 0.1 | 10 | 1.8 (1.0-2.5) | 0.1 | 10 | 3.2 (-0.7-10.1) | 0.6 |
| LEG | controls | 12 | 4.6 (4.5-4.8) |  | | |  | | |  | | |  | | |
|  | patients: NA | 34 | 27.4 (15.8-36.8)**^**^** | 26 | 3.0 (1.1-7.1) | 0.7 | 27 | 2.3 (0.2-6.0) | 0.6 | 28 | 2.9 (-0.2-4.7) | 0.5 | 29 | 8.4 (3.3-14.7) | **1.0** |
|  | patients: A | 14 | 73.6 (60.9-78.9)**^**^** | 12 | 0.8 (-0.7-1.9) | 0.2 | 11 | 1.4 (-3.5-2.5) | -0.1 | 10 | 0.0 (-2.2-0.7) | -0.3 | 10 | 0.5 (-5.3-3.0) | -0.1 |
|  |  |  | |  | | |  | | |  | | |  | | |
| **segment** | **group** | **cCSA at BL (cm^2^)** | | **ΔcCSA between BL and Y1 (%)** | | | **ΔcCSA between Y1 and Y2 (%)** | | | **ΔcCSA between Y2 and Y3 (%)** | | | **ΔcCSA BL-Y3 (%)** | | |
|  |  | ***n*** | **Med (IQD)** | ***n*** | **Med (IQD)** | **SRM** | ***n*** | **Med (IQD)** | **SRM** | ***n*** | **Med (IQD)** | **SRM** | ***n*** | **Med (IQD)** | **SRM** |
| THIGH | controls | 12 | 97 (88-118) |  | | |  | | |  | | |  | | |
|  | patients: A | 34 | 40 (29-64)**^**^** | 26 | -3.8 (-12.5-12.0) | -0.1 | 26 | -9.3 (-18.1- -3.1) | -0.3 | 30 | -10.4 (-16.4- -1.5) | -0.4 | 30 | -11.0 (-31.2- -0.2) | -0.5 |
|  | patients: NA | 14 | 13 (8-19)**^**^** | 12 | 5.4 (-6.9-17.4) | 0.3 | 12 | 0.7 (-10.0-31.2) | 0.3 | 10 | -6.3 (-16.4-14.5) | 0.2 | 10 | 3.5 (-17.3-38.4) | 0.4 |
| LEG | controls | 12 | 50 (49-55) |  | | |  | | |  | | |  | | |
|  | patients: A | 34 | 19 (14-26)**^**^** | 26 | -2.3 (-15.4-11.2) | -0.1 | 27 | -9.5 (-20.5- -1.0) | -0.3 | 28 | -4.0 (-14.9-4.8) | 0.1 | 29 | -12.8 (-30.0- -0.2) | -0.5 |
|  | patients: NA | 14 | 9 (7-12)**^**^** | 12 | 3.1 (-9.2-20.9) | 0.3 | 11 | -2.5 (-11.5-23.0) | 0.2 | 10 | 0.6 (-3.7-9.0) | 0.4 | 10 | 25.2 (-5.6-31.7) | 0.7 |

Between controls and patients: **P*<0.017; ***P*<0.001

A= ambulant; BL = baseline; cCSA = contractile cross-sectional area; FF = fat fraction (in %); IQD = interquartile distance; MED = median; *n* = number of patients; NA = non-ambulant; SRM = standardized response mean; Y1 = year-1; Y2 = year-2; Y3 = year-3; ΔcCSA = change in cCSA (in %, relative); ΔFF = change in FF (in %, absolute)

**Supplementary Table 3 Individual thigh muscle baseline FF values in controls and patients, and annual and 3-year ΔFF values in patients**

| **muscle** | **group** | **FF at BL** | | **ΔFF between BL and Y1** | | | **ΔFF between Y1 and Y2** | | | **ΔFF between Y2 and Y3** | | | **ΔFF after 3 years** | | |
| --- | --- | --- | --- | --- | --- | --- | --- | --- | --- | --- | --- | --- | --- | --- | --- |
|  |  | ***n*** | **Med (IQD)** | ***n*** | **Med (IQD)** | **SRM** | ***n*** | **Med (IQD)** | **SRM** | ***n*** | **Med (IQD)** | **SRM** | ***n*** | **Med (IQD)** | **SRM** |
| VL | controls | 12 | 3.1 (2.8-4.2)**^**^** |  | | |  | | |  | | |  | | |
|  | patients: A | 34 | 26.4 (7.6-43.1) | 26 | 2.9 (1.3-6.3) | 0.7 | 26 | 3.7 (1.0-7.3) | **1.0** | 30 | 2.3 (0.6-5.0) | 0.7 | 30 | 10.1 (4.1-14.6) | **0.9** |
|  | patients: NA | 14 | 71.2 (62.0-83.5) | 12 | 2.2 (-1.2-4.4) | 0.5 | 12 | 0.2 (-1.9-1.8) | 0.1 | 10 | 1.0 (-0.2-2.1) | -0.1 | 10 | 4.6 (0.6-7.0) | 0.3 |
| VM | controls | 12 | 1.9 (1.4-4.4)**^**^** |  | | |  | | |  | | |  | | |
|  | patients: A | 34 | 31.3 (12.1-45.1) | 26 | 2.6 (0.2-5.7) | **0.9** | 26 | 2.9 (0.9-7.3) | **0.9** | 30 | 2.4 (0.2-5.9) | **0.9** | 30 | 8.3 (3.3-15.2) | **1.1** |
|  | patients: NA | 14 | 65.6 (55.6-83.2) | 12 | 1.1 (-1.1-3.6) | 0.4 | 12 | 1.2 (-0.4-3.2) | 0.2 | 10 | 1.1 (-0.3-2.0) | 0.1 | 10 | 4.0 (-0.8-6.8) | 0.4 |
| VI | controls | 12 | 3.1 (2.8-3.7)**^**^** |  |  |  |  |  |  |  |  |  |  |  |  |
|  | patients: A | 34 | 28.6 (9.1-47.2) | 26 | 3.1 (1.0-6.0) | **1.0** | 26 | 1.9 (0.8-5.7) | 0.6 | 30 | 2.9 (0.6-6.2) | **0.8** | 30 | 7.6 (3.2-14.0) | **1.0** |
|  | patients: NA | 14 | 73.3 (60.6-84.8) | 12 | 1.1 (-0.5-4.4) | 0.5 | 12 | 0.4 (-1.4-2.3) | 0.0 | 10 | 1.0 (-2.8-2.4) | -0.1 | 10 | 3.3 (0.1-6.7) | 0.4 |
| BF | controls | 12 | 3.4 (2.6-4.2)**^**^** |  |  |  |  |  |  |  |  |  |  |  |  |
|  | patients: A | 34 | 29.7 (11.6-54.7) | 26 | 3.4 (0.8-7.0) | 0.6 | 26 | 3.2 (0.7-7.0) | **0.8** | 30 | 2.4 (0.1-5.9) | 0.6 | 30 | 9.7 (1.9-19.8) | **0.9** |
|  | patients: NA | 14 | 75.9 (70.3-84.1) | 12 | 0.5 (-1.4-1.7) | 0.1 | 12 | 0.8 (-2.5-2.8) | 0.0 | 10 | 1.2 (-0.6-3.3) | 0.2 | 10 | 3.3 (-1.2-7.0) | 0.5 |
| SM | controls | 12 | 3.3 (2.5-4.8)**^**^** |  |  |  |  |  |  |  |  |  |  |  |  |
|  | patients: A | 34 | 42.3 (23.6-70.4) |  | 3.5 (0.8-8.3) | **0.9** | 26 | 3.4 (0.0-5.9) | 0.5 | 30 | 2.1 (-0.6-5.9) | 0.6 | 30 | 9.1 (2.7-18.6) | **1.0** |
|  | patients: NA | 14 | 78.6 (69.0-85.8) | 26 | 0.0 (-1.8-4.2) | 0.2 | 12 | -0.1 (-1.7-3.1) | -0.1 | 10 | 0.9 (-1.5-2.7) | 0.1 | 10 | 1.6 (0.1-4.8) | 0.2 |
| ST | controls | 12 | 3.8 (2.9-4.7)**^**^** |  |  |  |  |  |  |  |  |  |  |  |  |
|  | patients: A | 34 | 28.9 (14.4-54.6) | 26 | 2.8 (0.2-10.9) | 0.7 | 26 | 2.4 (0.2-7.6) | 0.6 | 30 | 1.7 (-0.1-5.8) | 0.5 | 30 | 6.5 (1.7-20.4) | **0.8** |
|  | patients: NA | 14 | 72.2 (60.6-79.7) | 12 | 0.8 (-1.6-5.0) | 0.4 | 12 | -0.6 (-3.7-3.5) | -0.1 | 10 | 0.9 (-0.9-3.6) | 0.1 | 10 | 2.6 (-0.7-4.7) | 0.4 |
| AM | controls | 12 | 2.5 (2.3-4.6)**^**^** |  |  |  |  |  |  |  |  |  |  |  |  |
|  | patients: A | 34 | 38.5 (19.7-59.3) | 26 | 3.9 (0.3-8.1) | 0.6 | 26 | 2.3 (0.4-10.4) | 0.5 | 30 | 1.6 (-0.4-8.2) | 0.5 | 30 | 9.3 (2.2-18.0) | **0.9** |
|  | patients: NA | 14 | 75.7 (66.1-83.8) | 12 | 0.3 (-2.1-9.7) | 0.4 | 12 | 2.8 (-0.5-5.1) | 0.6 | 10 | 1.0 (-4.6-1.9) | 0.0 | 10 | 3.2 (1.8-13.1) | 0.7 |
| GRA | controls | 12 | 4.2 (3.1-5.8)**^**^** |  |  |  |  |  |  |  |  |  |  |  |  |
|  | patients: A | 34 | 7.4 (6.2-21.2) | 26 | 2.2 (0.8-6.7) | 0.7 | 26 | 0.0 (-2.0-3.2) | 0.2 | 30 | 0.7 (-0.6-3.2) | 0.2 | 30 | 3.3 (0.1-9.1) | 0.7 |
|  | patients: NA | 14 | 62.7 (48.6-75.1) | 12 | 3.5 (-0.9-6.7) | 0.5 | 12 | 0.5 (-3.3-4.8) | 0.1 | 10 | 2.0 (0.6-5.9) | 0.3 | 10 | 5.3 (-1.8-12.4) | 0.6 |
| SAR | controls | 12 | 3.9 (3.0-5.1)**^**^** |  | | |  | | |  | | |  | | |
|  | patients: A | 34 | 12.2 (8.4-23.2) | 26 | 2.8 (0.4-6.1) | 0.7 | 26 | 0.8 (-0.7-2.8) | 0.4 | 30 | 0.8 (-0.4-4.9) | 0.5 | 30 | 5.9 (0.5-11.4) | **0.8** |
|  | patients: NA | 14 | 69.6 (41.1-80.0) | 12 | 3.0 (-0.7-8.0) | 0.7 | 12 | 1.0 (-4.1-5.6) | 0.1 | 10 | 1.2 (-0.1-3.7) | 0.1 | 10 | 5.4 (-3.1-10.3) | 0.5 |

Between controls and patients: **P*<0.017; ***P*<0.001

A= ambulant; AM= adducor magnus; BF = biceps femoris; BL = baseline; FF = fat fraction (in %); GRA = gracilis; IQD = interquartile distance; MED = median; *n* = number of patients; NA = non-ambulant; SAR= Sartorius; SM = semimembranosus; SRM = standardized response mean; ST = semitendinosus; VI = vastus intermedius; VL = vastus lateralis; VM = vastus medialis; Y1 = year-1; Y2 = year-2; Y3 = year-3; ΔFF = change in FF (in %, absolute)

**Supplementary Table 4 Individual leg muscle baseline FF values in controls and patients, and annual and 3-year ΔFF values in patients**

| **muscle** | **group** | **FF at BL** | | **ΔFF between BL and Y1** | | | **ΔFF between Y1 and Y2** | | | **ΔFF between Y2 and Y3** | | | **ΔFF after 3 years** | | |
| --- | --- | --- | --- | --- | --- | --- | --- | --- | --- | --- | --- | --- | --- | --- | --- |
|  |  | ***n*** | **Med (IQD)** | ***n*** | **Med (IQD)** | **SRM** | ***n*** | **Med (IQD)** | **SRM** | ***n*** | **Med (IQD)** | **SRM** | ***n*** | **Med (IQD)** | **SRM** |
| ED | controls | 12 | 4.3 (3.3-6.3)**^**^** |  | | |  | | |  | | |  | | |
|  | patients: A | 34 | 23.4 (9.3-48.9) | 26 | 1.9 (0.2-5.5) | 0.6 | 27 | 1.6 (-0.5-3.6) | 0.4 | 28 | 1.7 (-0.1-5.5) | 0.3 | 29 | 6.5 (1.2-14.7) | **0.8** |
|  | patients: NA | 14 | 78.7 (64.4-86.6) | 12 | 0.1 (-1.1-2.0) | 0.0 | 11 | 1.1 (-3.6-2.7) | -0.2 | 10 | -0.3 (-2.3-2.4) | -0.2 | 10 | -1.9 (-9.0-3.4) | -0.4 |
| TA | controls | 12 | 3.6 (2.3-6.4)**^**^** |  | | |  | | |  | | |  | | |
|  | patients: A | 34 | 10.9 (6.0-23.5) | 26 | 2.1 (0.8-6.1) | 0.5 | 27 | 2.3 (0.3-6.2) | 0.5 | 28 | 1.8 (-0.5-5.9) | 0.3 | 29 | 7.9 (1.6-14.4) | **1.0** |
|  | patients: NA | 14 | 69.2 (52.9-77.0) | 12 | 0.6 (-0.5-5.0) | 0.5 | 11 | 0.6 (-3.6-2.3) | -0.1 | 10 | 0.3 (-1.3-2.3) | -0.1 | 10 | 0.4 (-4.1-4.7) | 0.1 |
| TP | controls | 12 | 4.0 (2.9-4.3)**^**^** |  |  |  |  |  |  |  |  |  |  |  |  |
|  | patients: A | 34 | 11.9 (6.2-22.9) | 26 | 1.5 (0.0-3.8) | 0.5 | 27 | 0.5 (-0.5-4.1) | 0.3 | 28 | 1.2 (-0.4-5.3) | 0.5 | 29 | 3.0 (0.5-18.6) | **0.8** |
|  | patients: NA | 14 | 69.8 (44.1-75.6) | 12 | 1.7 (-1.9-3.7) | 0.3 | 11 | -0.2 (-3.0-2.2) | -0.2 | 10 | 0.8 (-0.1-3.0) | 0.0 | 10 | 2.9 (-2.3-5.4) | 0.1 |
| PER | controls | 12 | 5.5 (4.6-7.9)**^**^** |  |  |  |  |  |  |  |  |  |  |  |  |
|  | patients: A | 34 | 13.5 (7.7-29.3) | 26 | 3.2 (0.4-7.3) | 0.6 | 27 | 1.6 (0.2-5.3) | 0.4 | 28 | 2.2 (-0.9-7.7) | 0.3 | 29 | 8.3 (2.8-17.5) | **0.9** |
|  | patients: NA | 14 | 65.9 (45.1-76.8) | 12 | 0.0 (-2.4-1.1) | 0.1 | 11 | 0.0 (-2.1-1.3) | -0.1 | 10 | -0.8 (-3.5-1.2) | -0.3 | 10 | -1.6 (-6.3-1.3) | -0.1 |
| SOL | controls | 12 | 3.6 (2.8-4.8)**^**^** |  |  |  |  |  |  |  |  |  |  |  |  |
|  | patients: A | 34 | 33.2 (19.1-49.3) | 26 | 3.3 (-0.9-6.2) | 0.5 | 27 | 1.7 (-1.2-10.4) | 0.5 | 28 | 1.7 (-1.6-5.3) | 0.3 | 29 | 7.7 (1.6-16.5) | **0.9** |
|  | patients: NA | 14 | 75.2 (65.9-81.1) | 12 | -0.3 (-2.3-1.3) | -0.1 | 11 | 0.3 (-4.2-2.8) | -0.1 | 10 | -1.1 (-2.0-1.2) | -0.3 | 10 | -0.3 (-4.7-1.1) | -0.4 |
| GM | controls | 12 | 3.1 (2.7-4.3)**^**^** |  |  |  |  |  |  |  |  |  |  |  |  |
|  | patients: A | 34 | 47.8 (25.3-64.3) | 26 | 2.4 (-0.9-10.1) | 0.4 | 27 | 4.1 (-1.0-9.3) | 0.5 | 28 | 1.0 (-2.8-4.0) | 0.0 | 29 | 7.1 (0.2-18.8) | 0.7 |
|  | patients: NA | 14 | 75.8 (62.2-78.7) | 12 | 1.2 (-1.1-2.8) | 0.4 | 11 | -1.4 (-4.5-1.6) | -0.2 | 10 | 0.0 (-4.0-1.5) | 0.0 | 10 | 0.6 (-6.0-2.8) | -0.1 |
| GL | controls | 12 | 2.7 (2.4-3.7)**^**^** |  |  |  |  |  |  |  |  |  |  |  |  |
|  | patients: A | 34 | 26.9 (13.2-47.3) | 26 | 3.7 (0.1-10.4) | 0.5 | 27 | 2.7 (-0.2-5.8) | 0.4 | 28 | 0.8 (-1.9-5.1) | 0.1 | 29 | 7.2 (1.3-17.6) | **0.8** |
|  | patients: NA | 14 | 76.5 (56.0-82.3) | 12 | 2.2 (-0.1-5.2) | 0.6 | 11 | -1.5 (-4.3-1.3) | -0.3 | 10 | -0.2 (-3.9-1.6) | -0.3 | 10 | 0.7 (-4.7-2.5) | 0.0 |

Between controls and patients: **P*<0.017; ***P*<0.001

A= ambulant; BL = baseline; ED = extensor digitorum longus; FF = fat fraction (in %) GL = gastrocnemius lateralis; GM = gastrocnemius medialis; IQD = interquartile distance; MED = median; *n* = number of patients; NA = non-ambulant; PER = peroneus longus; SOL = soleus; SRM = standardized response mean; TA = tibialis anterior; TP = tibialis posterior; Y1 = year-1; Y2 = year-2; Y3 = year-3; ΔFF = change in FF (in %, absolute)

**Supplementary Table 5 Linear Mixed Model analysis for individual muscle FF data**

|  | **β** | **SE (β)** | **95% CI** | ***P*** |
| --- | --- | --- | --- | --- |
| Intercept | 21.7 | 5.6 | [10.6-32.7] | **<0.001** |
| Time=Y1 | 3.2 | 0.9 | [1.3-5.1] | **0.001** |
| Time=Y2 | 5.0 | 1.0 | [3.0-7.1] | **<0.001** |
| Time=Y3 | 5.7 | 1.2 | [3.4-8.1] | **<0.001** |
| Group=NA | 27.7 | 5.5 | [16.7-38.8] | **<0.001** |
| Time=Y1*Group=NA | -1.9 | 1.7 | [-5.2-1.5] | 0.281 |
| Time=Y2*Group=NA | -5.6 | 1.7 | [-8.9- -2.3] | **0.001** |
| Time=Y3*Group=NA | -5.9 | 1.8 | [-9.4- -2.4] | **0.001** |
| Muscle=VL | 4.6 | 1.4 | [1.9-7.3] | **0.001** |
| Muscle=VI | 5.3 | 1.4 | [2.5-8.1] | **<0.001** |
| Muscle=BF | 1.1 | 1.4 | [-1.7-3.9] | 0.444 |
| Muscle=SM | 12.3 | 1.4 | [9.5-15.1] | **<0.001** |
| Muscle=ST | 6.1 | 1.4 | [3.3-8.9] | **<0.001** |
| Muscle=AM | 10.2 | 1.4 | [7.5-13.0] | **<0.001** |
| Muscle=GRA | -7.8 | 1.4 | [-10.6- -5.0] | **<0.001** |
| Muscle=SAR | 2.2 | 1.4 | [-0.6-5.0] | 0.124 |
| Muscle=ED | -1.4 | 1.4 | [-4.2-1.4] | 0.337 |
| Muscle=TA | -5.4 | 1.4 | [-8.3- -2.6] | **<0.001** |
| Muscle=TP | -0.5 | 1.4 | [-3.3-2.4] | 0.750 |
| Muscle=PER | 5.4 | 1.4 | [2.6-8.2] | **<0.001** |
| Muscle=SOL | 2.6 | 1.4 | [-0.3-5.3] | 0.076 |
| Muscle=GM | 11.8 | 1.4 | [9.0-14.6] | **<0.001** |
| Muscle=GL | 11.3 | 1.4 | [8.5-14.1] | **<0.001** |
| Site=Paris | -5.8 | 5.2 | [-16.2-4.6] | 0.265 |
| Years since onset symptoms | 0.6 | 0.3 | [0.1-1.1] | 0.024 |
| BMI | 0.03 | 0.1 | [-0.2-0.3] | 0.839 |
| *(VPC (%)* | *60.6* |  | *[52.0-68.6])* |  |

^a^Estimate for VM of ambulant patients (group=A) in site=Newcastle at baseline

A= ambulant; AM= adducor magnus; BF = biceps femoris; CI = confidence interval; ED = extensor digitorum longus; FF = fat fraction (in %); GL = gastrocnemius lateralis; GM = gastrocnemius medialis; GRA = gracilis; ICC = intraclass correlation coefficient; NA = non-ambulant; PER = peroneus longus; SAR= Sartorius; SM = semimembranosus; SOL = soleus; ST = semitendinosus; SE = standard error; TA = tibialis anterior; TP = tibialis posterior; VI = vastus intermedius; VL = vastus lateralis; VM = vastus medialis VPC = variance partition coefficient; Y1 = year-1; Y2 = year-2; Y3 = year-3

**Supplementary Table 6 Global segment baseline water T_2_ values in controls and patients, and annual and 3-year average water T_2_ values in patients, in thigh and leg**

| **segment** | **group** | **water T_2_ (ms) at BL** | | **water T_2_ (ms) at Y1** | | | | **water T_2_ (ms) at Y2** | | | | **water T_2_ (ms) at Y3** | | | | **average water T_2_ (ms) over 3 years** | | | |
| --- | --- | --- | --- | --- | --- | --- | --- | --- | --- | --- | --- | --- | --- | --- | --- | --- | --- | --- | --- |
|  |  | ***n*** | **Med (IQD)** | ***n*** | **Med (IQD)** | **SDM** | **SRM** | ***n*** | **Med (IQD)** | **SDM** | **SRM** | ***n*** | **Med (IQD)** | **SDM** | **SRM** | ***n*** | **Med (IQD)** | **SDM** | **SRM** |
| THIGH | controls | 12 | 34.5 (34.0-35.2) |  | | | | | | | | | | | | | | | |
|  | patients: A | 35 | 40.0 (37.9-42.5)**^**^** | 28 | 40.8 (38.2-42.0) | **1.2** | 0.1 | 33 | 40.5 (38.2-42.8) | **3.1** | 0.2 | 30 | 39.9 (37.7-42.2) | **3.9** | -0.2 | 36 | 40.5 (38.0-42.0) | **1.2** | 0.1 |
|  | patients: NA | 14 | 38.0 (36.7-41.8)**^**^** | 12 | 36.6 (35.0-37.6) | 0.2 | -0.1 | 13 | 35.6 (33.6-37.7) | 0.2 | 0.2 | 11 | 36.3 (32.6-37.3) | 0.2 | 0.1 | 15 | 37.0 (36.0-38.0) | 0.4 | 0.4 |
| LEG | controls | 12 | 36.7 (35.9-37.5) |  | | | | | | | | | | | | | | | |
|  | patients: A | 36 | 39.5 (38.4-42.0)**^**^** | 29 | 41.0 (38.7-42.0) | **2.3** | 0.3 | 33 | 39.0 (37.8-41.6) | **1.0** | -0.1 | 32 | 39.1 (36.4-41.7) | **1.4** | 0.1 | 37 | 39.5 (38.2-42.9) | 0.6 | 0.0 |
|  | patients: NA | 15 | 38.5 (36.1-41.5) | 15 | 36.8 (35.4-40.5) | 0.0 | -0.1 | 14 | 37.2 (33.5-39.3) | 0.2 | 0.2 | 13 | 35.7 (33.0-39.2) | 0.0 | 0.0 | 16 | 38.0 (35.4-40.6) | 0.3 | 0.3 |

Between controls and patients: **P*<0.017; ***P*<0.001

A= ambulant; BL = baseline; IQD = interquartile distance; MED = median; *n* = number of patients; NA = non-ambulant; SDM = standardized difference mean; SRM = standardized response mean; Y1 = year-1; Y2 = year-2; Y3 = year-3

**Supplementary Table 7 Individual thigh muscle baseline water T_2_ values in controls and patients, and annual and 3-year average water T_2_ values in patients**

| **muscle** | **group** | **water T_2_ (ms) at BL** | | **water T_2_ (ms) at Y1** | | | | **water T_2_ (ms) at Y2** | | | | **water T_2_ (ms) at Y3** | | | | **average water T_2_ (ms) over 3 years** | | | |  |
| --- | --- | --- | --- | --- | --- | --- | --- | --- | --- | --- | --- | --- | --- | --- | --- | --- | --- | --- | --- | --- |
|  |  | ***n*^a^** | **Med (IQD)** | ***n*^a^** | **Med (IQD)** | **SDM** | **SRM** | ***n*^a^** | **Med (IQD)** | **SDM** | **SRM** | ***n*^a^** | **Med (IQD)** | **SDM** | **SRM** | ***n*^a^** | **Med (IQD)** | **SDM** | **SRM** | |
| VL | controls | 12 | 36.1 (34.6-37.8)* |  | | | |  | | | |  | | | |  | | | |  |
|  | patients: A | 35 | 42.4 (37.5-46.4) | 26 | 42.2 (37.7-49.5) | **2.0** | -0.2 | 33 | 41.7 (37.1-45.7) | **3.3** | 0.2 | 30 | 40.9 (37.8-44.1) | **1.5** | -0.1 | 36 | 42.0 (35.9-43.8) | **1.0** | -0.1 |  |
|  | patients: NA | 7 | 40.1 (35.9-41.4) | 7 | 37.6 (33.3-39.6) | -0.4 | -0.6 | 9 | 37.3 (33.9-40.9) | **2.3** | 0.6 | 9 | 37.1 (33.5-38.9) | 0.3 | -0.1 | 10 | 37.1 (34.4-40.2) | **-1.1** | **-1.1** |  |
| VM | controls | 12 | 34.4 (33.0-36.1)** |  | | | |  | | | |  | | | |  | | | |  |
|  | patients: A | 34 | 42.5 (37.9-45.7) | 25 | 42.7 (39.6-44.7) | **2.9** | -0.3 | 33 | 41.5 (37.0-44.9) | **2.4** | 0.0 | 28 | 42.0 (37.7-43.5) | **1.3** | -0.2 | 36 | 42.1 (37.7-44.1) | **1.0** | -0.4 |  |
|  | patients: NA | 6 | 37.6 (32.8-39.3) | 5 | 37.7 (36.9-38.3) | **3.6** | **-0.8** | 9 | 33.8 (30.0-36.5) | **-1.2** | -0.4 | 7 | 32.9 (31.0-37.6) | **2.9** | 0.3 | 11 | 36.4 (30.8-37.9) | **-1.3** | **-0.9** |  |
| VI | controls | 12 | 34.8 (33.1-36.3)** |  |  |  |  |  |  |  |  |  |  |  |  |  |  |  |  |  |
|  | patients: A | 35 | 41.2 (38.3-44.3) | 28 | 42.0 (38.3-43.3) | **2.3** | -0.1 | 33 | 41.1 (38.8-43.6) | **2.6** | 0.0 | 31 | 40.2 (37.5-41.8) | **1.9** | -0.3 | 36 | 41.3 (38.1-42.8) | **0.9** | -0.2 |  |
|  | patients: NA | 7 | 38.0 (37.1-40.8) | 6 | 36.4 (34.5-38.7) | 0.5 | -0.3 | 8 | 36.5 (31.5-37.8) | 0.0 | -0.1 | 8 | 36.1 (34.1-38.2) | **1.5** | 0.3 | 11 | 36.7 (34.1-38.8) | **-1.0** | -0.3 |  |
| BF | controls | 12 | 35.0 (34.2-35.9) |  |  |  |  |  |  |  |  |  |  |  |  |  |  |  |  |  |
|  | patients: A | 25 | 39.7 (37.6-44.0)**^##^** | 21 | 39.2 (36.9-43.3) | **1.5** | -0.3 | 28 | 39.1 (36.2-42.8) | **3.0** | 0.4 | 25 | 38.5 (35.2-42.4) | **0.8** | -0.7 | 32 | 38.3 (35.9-43.0) | **0.4** | -0.2 |  |
|  | patients: NA | 4 | 37.9 | 3 | 37.6 | 0.4 | -0.2 | 6 | 34.7 (32.6-36.1) | **2.0** | 0.7 | 3 | 32.6 | **-3.1** | -1.5 | 9 | 33.5 (32.5-38.0) | / |  |  |
| SM | controls | 12 | 35.2 (33.5-36.2) |  |  |  |  |  |  |  |  |  |  |  |  |  |  |  |  |  |
|  | patients: A | 23 | 40.3 (37.6-43.5) | 18 | 39.2 (37.1-44.0) | **0.9** | -0.3 | 27 | 37.3 (34.0-41.4) | **1.6** | -0.2 | 22 | 38.5 (35.6-42.2) | **1.1** | -0.2 | 30 | 37.9 (35.8-41.4) | **0.2** | -0.3 |  |
|  | patients: NA | 3 | 36.9 | 3 | 34.4 | **1.5** | -0.4 | 5 | 35.8 (31.8-37.6) | **1.1** | 0.4 | 4 | 31.5 | **-2.0** | -0.5 | 5 | 29.9 (31.0-35.6) | **-3.9** | **-1.7** |  |
| ST | controls | 12 | 33.5 (33.2-34.2)** |  |  |  |  |  |  |  |  |  |  |  |  |  |  |  |  |  |
|  | patients: A | 16 | 35.7 (34.9-38.5) | 14 | 37.6 (35.3-38.8) | **2.0** | 0.2 | 17 | 37.1 (33.1-39.2) | -0.4 | -0.2 | 22 | 35.9 (29.4-38.5) | -0.3 | -0.3 | 28 | 36.4 (29.7-37.9) | 0.5 | 0.0 |  |
|  | patients: NA | 0 | / | 2 | 33.3 | **/** | / | 3 | 26.8 | / | / | 1 | 23.7 | / | / | 4 | 29.9 | / | / |  |
| AM | controls | 12 | 34.1 (32.1-35.0)* |  |  |  |  |  |  |  |  |  |  |  |  |  |  |  |  |  |
|  | patients: A | 33 | 38.9 (37.2-41.4) | 26 | 39.7 (37.8-41.3) | **1.0** | 0.0 | 31 | 40.4 (37.9-43.2) | **2.4** | 0.2 | 29 | 39.9 (37.6-41.5) | 0.3 | -0.3 | 34 | 39.5 (37.9-41.8) | **1.7** | 0.1 |  |
|  | patients: NA | 5 | 38.0 (37.3-38.8) | 4 | 38.1 | **2.7** | 0.1 | 7 | 34.4 (31.1-37.4) | **-0.9** | **-1.4** | 5 | 35.4 (31.6-40.2) | **1.9** | 0.2 | 8 | 35.9 (32.1-37.9) | **-3.7** | **-0.8** |  |
| GRA | controls | 12 | 32.4 (30.6-34.0)** |  |  |  |  |  |  |  |  |  |  |  |  |  |  |  |  |  |
|  | patients: A | 33 | 37.4 (35.4-39.8) | 26 | 37.5 (36.0-40.0) | **1.9** | 0.0 | 32 | 38.7 (35.9-41.2) | **2.8** | 0.6 | 28 | 38.1 (35.9-42.2) | **2.3** | 0.2 | 36 | 38.0 (35.8-40.6) | **2.7** | 0.5 |  |
|  | patients: NA | 6 | 39.1 (36.5-39.9) | 5 | 37.6 (35.5-38.6) | 0.6 | **-0.8** | 8 | 37.7 (34.5-38.5) | -0.6 | -0.4 | 4 | 40.2 | **1.7** | 0.2 | 9 | 37.8 (35.1-39.8) | -0.7 | -0.5 |  |
| SAR | controls | 12 | 35.0 (33.4-36.0)** |  |  |  |  |  |  |  |  |  |  |  |  |  |  |  |  |  |
|  | patients: A | 32 | 39.2 (36.3-41.2) | 25 | 39.4 (36.0-41.1) | **1.8** | -0.1 | 31 | 39.6 (35.9-41.8) | **2.3** | 0.2 | 27 | 38.5 (35.5-41.9) | **1.9** | 0.0 | 34 | 39.2 (35.8-41.0) | **1.7** | 0.1 |  |
|  | patients: NA | 4 | 37.6 | 6 | 36.8 (35.0-37.9) | **1.7** | 0.4 | 7 | 34.4 (33.2-37.2) | **-1.1** | -0.3 | 6 | 37.1 (31.0-39.7) | **4.8** | 0.4 | 8 | 34.7 (31.6-38.4) | **1.0** | 0.3 |  |

Between controls and patients: **P*<0.017; ***P*<0.001

^a^When less than 5 patients, the average or singular value is given.

A= ambulant; AM= adducor magnus; BF = biceps femoris; BL = baseline; GRA = gracilis; IQD = interquartile distance; MED = median; *n* = number of patients; NA = non-ambulant; SAR= Sartorius; SM = semimembranosus; ST = semitendinosus; SDM = standardized difference mean; SRM = standardized response mean; VI = vastus intermedius; VL = vastus lateralis; VM = vastus medialis; Y1 = year-1; Y2 = year-2; Y3 = year-3

**Supplementary Table 8 Individual leg muscle baseline water T_2_ values in controls and patients, and annual and 3-year average water T_2_ values in patients**

| **muscle** | **group** | **water T_2_ (ms) at BL** | | **water T_2_ (ms) at Y1** | | | | **water T_2_ (ms) at Y2** | | | | **water T_2_ (ms) at Y3** | | | | **average water T_2_ (ms) over 3 years** | | | |
| --- | --- | --- | --- | --- | --- | --- | --- | --- | --- | --- | --- | --- | --- | --- | --- | --- | --- | --- | --- |
|  |  | ***n*^a^** | **Med (IQD)** | ***n*^a^** | **Med (IQD)** | **SDM** | **SRM** | ***n*^a^** | **Med (IQD)** | **SDM** | **SRM** | ***n*^a^** | **Med (IQD)** | **SDM** | **SRM** | ***n*** | **Med (IQD)** | **SDM** | **SRM** |
| ED | controls | 12 | 36.0 (35.7-36.6)** |  | | | |  | | | |  | | | |  | | | |
|  | patients: A | 34 | 40.2 (38.0-42.7) | 27 | 41.5 (39.3-43.2) | **-1.1** | -0.3 | 33 | 40.2 (37.4-42.4) | **-1.3** | -0.5 | 32 | 40.7 (38.2-44.1) | **1.3** | 0.0 | 36 | 40.5 (38.9-43.0) | 0.2 | -0.1 |
|  | patients: NA | 9 | 40.3 (31.7-45.5) | 11 | 37.7 (27.3-41.0) | **-1.2** | -0.3 | 9 | 35.9 (30.5-38.3) | **-2.1** | -0.5 | 9 | 36.6 (28.7-42.3) | 0.2 | 0.0 | 12 | 36.8 (33.1-40.8) | -0.2 | -0.1 |
| TA | controls | 12 | 36.6 (36.0-37.0)** |  | | | |  | | | |  | | | |  | | | |
|  | patients: A | 34 | 40.5 (38.0-43.9) | 27 | 40.3 (38.6-43.6) | **1.5** | -0.2 | 33 | 40.7 (37.1-43.9) | **1.9** | 0.2 | 31 | 39.0 (36.8-43.6) | **1.0** | 0.0 | 36 | 40.4 (37.9-43.8) | **0.8** | 0.0 |
|  | patients: NA | 11 | 36.0 (35.3-40.8) | 12 | 37.8 (35.1-40.8) | -0.5 | -0.3 | 12 | 36.4 (34.6-39.7) | -0.1 | -0.1 | 13 | 34.4 (31.6-36.7) | **-1.3** | -0.4 | 15 | 36.5 (33.7-39.7) | **-1.6** | -0.3 |
| TP | controls | 12 | 36.8 (36.3-37.6)** |  | | | | | | | | | | | | | | | |
|  | patients: A | 32 | 40.2 (37.5-43.1) | 27 | 39.7 (36.9-42.5) | **1.4** | 0.0 | 33 | 40.5 (37.7-43.2) | **2.6** | 0.4 | 33 | 40.1 (37.2-43.4) | **1.5** | -0.1 | 36 | 40.1 (37.9-43.1) | **1.3** | 0.1 |
|  | patients: NA | 8 | 43.0 (38.7-49.0) | 7 | 39.0 (38.0-41.5) | 0.6 | -0.4 | 8 | 40.4 (39.6-44.5) | **5.4** | **1.8** | 7 | 38.8 (36.5-39.9) | 0.3 | -0.6 | 11 | 40.2 (39.1-44.9) | **-3.7** | -0.5 |
| PER | controls | 12 | 37.2 (36.8-37.4) |  | | | | | | | | | | | | | | | |
|  | patients: A | 31 | 38.4 (35.7-43.5) | 22 | 39.3 (36.5-42.6) | **0.3** | -0.3 | 32 | 38.2 (32.1-42.5) | 0.1 | -0.1 | 31 | 37.3 (32.8-42.2) | **1.2** | 0.1 | 36 | 37.8 (32.3-43.3) | 0.3 | 0.0 |
|  | patients: NA | 4 | 37.6 | 8 | 35.5 (31.4-41.5) | **-1.9** | **-1.1** | 9 | 33.3 (30.1-44.5) | 0.6 | 0.6 | 9 | 34.5 (33.2-39.9) | -0.2 | 0.3 | 12 | 33.9 (32.2-44.9) | **-4.3** | -0.6 |
| SOL | controls | 12 | 37.2 (36.8-37.4) |  | | | | | | | | | | | | | | | |
|  | patients: A | 31 | 37.3 (34.0-41.3) | 23 | 37.7 (33.1-43.1) | **1.1** | 0.2 | 32 | 36.3 (32.2-40.1) | **-1.3** | -0.3 | 29 | 36.8 (31.0-41.8) | **1.1** | 0.2 | 35 | 36.2 (31.6-42.1) | **0.8** | 0.1 |
|  | patients: NA | 6 | 37.0 (32.6-42.6) | 6 | 34.2 (31.8-36.0) | **1.7** | 0.3 | 9 | 31.6 (28.5-33.9) | -0.7 | 0.1 | 8 | 32.3 (31.0-35.7) | 0.2 | **0.8** | 12 | 33.4 (30.6-37.6) | **2.4** | 0.7 |
| GM | controls | 12 | 37.8 (36.6-38.4) |  | | | | | | | | | | | | | | | |
|  | patients: A | 21 | 35.3 (31.9-38.9) | 19 | 37.3 (34.4-40.5) | **1.6** | 0.4 | 30 | 34.2 (30.3-38.0) | -0.5 | -0.1 | 27 | 34.2 (32.6-40.7) | **1.9** | 0.4 | 34 | 34.9 (31.9-38.9) | **1.1** | 0.1 |
|  | patients: NA | 4 | 37.8 | 5 | 36.1 (32.3-37.1) | **/** | / | 10 | 36.3 (30.9-39.9) | **5.7** | **2.0** | 7 | 38.5 (34.8-40.0) | **3.1** | 0.6 | 13 | 37.0 (32.9-39.7) | **2.2** | 0.2 |
| GL | controls | 12 | 37.9 (37.4-38.4) |  | | | | | | | | | | | | | | | |
|  | patients: A | 25 | 38.2 (34.2-40.8) | 18 | 36.7 (34.2-42.4) | **1.1** | 0.2 | 28 | 36.1 (31.0-39.9) | -0.4 | -0.1 | 26 | 37.2 (30.6-39.7) | -0.4 | -0.1 | 34 | 36.2 (31.8-40.2) | **-0.8** | -0.1 |
|  | patients: NA | 1 | 38.3 | 4 | 38.3 | / | / | 7 | 33.0 (30.5-38.2) | **1.4** | 0.2 | 3 | 41.2 | / | / | 10 | 38.4 (33.7-41.6) | / | / |

Between controls and patients: **P*<0.017; ***P*<0.001

^a^When less than 5 patients, the average or singular value is given.

A= ambulant; BL = baseline; ED = extensor digitorum longus; GL = gastrocnemius lateralis; GM = gastrocnemius medialis; IQD = interquartile distance; MED = median; *n* = number of patients; NA = non-ambulant; PER = peroneus longus; SDM = standardized difference mean; SOL = soleus; SRM = standardized response mean; TA = tibialis anterior; TP = tibialis posterior; Y1 = year-1; Y2 = year-2; Y3 = year-3

**Supplementary Table 9 Linear Mixed Model analysis for individual muscle water T_2_ data**

|  | **β** | **SE (β)** | **95% CI** | ***P*** |
| --- | --- | --- | --- | --- |
| Intercept^a^ | 43.1 | 0.8 | [41.4-44.7] | **<0.001** |
| Time=Y1 | -0.1 | 0.4 | [-0.8-0.7] | 0.894 |
| Time=Y2 | 0.03 | 0.4 | [-0.7-0.8] | 0.933 |
| Time=Y3 | 0.2 | 0.4 | [-0.6-1.0] | 0.677 |
| Group=NA | 0.1 | 1.2 | [-2.3-2.6] | 0.934 |
| Time=Y1*Group=NA | -1.9 | 0.9 | [-3.7- -0.1] | 0.036 |
| Time=Y2*Group=NA | -2.3 | 0.9 | [-4.0- -0.6] | **0.009** |
| Time=Y3*Group=NA | -1.8 | 0.9 | [-3.5- -0.05] | 0.044 |
| Muscle=VL | -0.2 | 0.4 | [-0.9-0.6] | 0.632 |
| Muscle=VI | -0.4 | 0.4 | [-1.2-0.5] | 0.384 |
| Muscle=BF | -1.3 | 0.5 | [-2.2- -0.4] | **0.007** |
| Muscle=SM | -2.7 | 0.5 | [-3.7- -1.7] | **<0.001** |
| Muscle=ST | -2.7 | 0.5 | [-3.8- -1.7] | **<0.001** |
| Muscle=AM | -0.6 | 0.4 | [-1.5-0.2] | 0.124 |
| Muscle=GRA | -2.4 | 0.5 | [-3.3- -1.4] | **<0.001** |
| Muscle=SAR | -1.9 | 0.5 | [-2.9- -1.0] | **<0.001** |
| Muscle=ED | -0.9 | 0.5 | [-1.8- -0.02] | 0.045 |
| Muscle=TA | -0.8 | 0.5 | [-1.7-0.1] | 0.085 |
| Muscle=TP | -0.6 | 0.5 | [-1.5-0.3] | 0.170 |
| Muscle=PER | -2.8 | 0.5 | [-3.7- -1.8] | **<0.001** |
| Muscle=SOL | -2.1 | 0.5 | [-3.0- -1.1] | **<0.001** |
| Muscle=GM | -3.2 | 0.5 | [-4.1- -2.2] | **<0.001** |
| Muscle=GL | -3.7 | 0.5 | [-4.7- -2.7] | **<0.001** |
| Site=Paris | -1.6 | 1.0 | [-3.6-0.4] | 0.122 |
| Years since onset symptoms | -0.04 | 0.05 | [-0.1-0.1] | 0.404 |
| FF | -0.03 | 0.01 | [-0.04- -0.02] | **<0.001** |
| *(VPC (%)* | *32.4* |  | *[24.8-41.1])* |  |

^a^Estimate for VM of ambulant patients (group=A) in site=Newcastle at baseline

A = ambulant; AM= adducor magnus; BF = biceps femoris; CI = confidence interval; ED = extensor digitorum longus; FF = fat fraction (in %); GL = gastrocnemius lateralis; GM = gastrocnemius medialis; GRA = gracilis; ICC = intraclass correlation coefficient; NA = non-ambulant; PER = peroneus longus; SAR= Sartorius; SM = semimembranosus; SOL = soleus; ST = semitendinosus; SE = standard error; TA = tibialis anterior; TP = tibialis posterior; VI = vastus intermedius; VL = vastus lateralis; VM = vastus medialis; VPC = variance partition coefficient; Y1 = year-1; Y2 = year-2; Y3 = year-3

**Supplementary Table 10 Baseline ^31^P MRS values in controls and patients, and annual and 3-year average ^31^P MRS values values in patients, in the anterior leg compartment**

| **group** | **pH_w_ BL** | | **pH_w_ at Y1** | | | | **pH_w_ at Y2** | | | | **pH_w_ at Y3** | | | | **average pH_w_ over 3 years** | | | |
| --- | --- | --- | --- | --- | --- | --- | --- | --- | --- | --- | --- | --- | --- | --- | --- | --- | --- | --- |
|  | ***n*** | **Med (IQD)** | ***n*** | **Med (IQD)** | **SDM** | **SRM** | ***n*** | **Med (IQD)** | **SDM** | **SRM** | ***n*** | **Med (IQD)** | **SDM** | **SRM** | ***n*** | **Med (IQD)** | **SDM** | **SRM** |
| controls | 9 | 7.03 (7.01-7.08) |  | | | | | | | | | | | | | | | |
| patients: A | 19 | 7.08 (7.07-7.11) | 11 | 7.09 (7.04-7.10) | **1.6** | 0.0 | 16 | 7.07 (7.05-7.14) | **1.7** | 0.3 | 15 | 7.09 (7.03-7.13) | **1.4** | 0.2 | 19 | 7.08 (7.05-7.12) | **1.3** | 0.2 |
| **group** | **PDE/γATP BL** | | **PDE/γATP at Y1** | | | | **PDE/γATP at Y2** | | | | **PDE/γATP at Y3** | | | | **average PDE/γATP over 3 years** | | | |
|  | ***n*** | **Med (IQD)** | ***n*** | **Med (IQD)** | **SDM** | **SRM** | ***n*** | **Med (IQD)** | **SDM** | **SRM** | ***n*** | **Med (IQD)** | **SDM** | **SRM** | ***n*** | **Med (IQD)** | **SDM** | **SRM** |
| controls | 9 | 0.20 (0.18-0.21) |  | | | | | | | | | | | | | | | |
| patients: A | 19 | 0.44 (0.37-0.53)^**^ | 11 | 0.47 (0.40-0.50) | **2.5** | 0.0 | 15 | 0.43 (0.41-0.51) | **2.4** | 0.1 | 13 | 0.47 (0.40-0.61) | **2.0** | 0.1 | 19 | 0.46 (0.37-0.51) | **2.7** | 0.3 |
| **group** | **PME/γATP BL** | | **PME/γATP at Y1** | | | | **PME/γATP at Y2** | | | | **PME/γATP at Y3** | | | | **average PME/γATP over 3 years** | | | |
|  | ***n*** | **Med (IQD)** | ***n*** | **Med (IQD)** | **SDM** | **SRM** | ***n*** | **Med (IQD)** | **SDM** | **SRM** | ***n*** | **Med (IQD)** | **SDM** | **SRM** | ***n*** | **Med (IQD)** | **SDM** | **SRM** |
| controls | 9 | 0.17 (0.16-0.21) |  | | | | | | | | | | | | | | | |
| patients: A | 19 | 0.33 (0.25-0.38)^**^ | 11 | 0.32 (0.27-0.39) | **1.7** | 0.4 | 16 | 0.37 (0.28-0.46) | **1.4** | 0.4 | 15 | 0.30 (0.22-0.43) | **1.3** | -0.1 | 19 | 0.33 (0.25-0.46) | **1.5** | 0.4 |
| **group** | **PCr/γATP BL** | | **PCr/γATP at Y1** | | | | **PCr/γATP at Y2** | | | | **PCr/γATP at Y3** | | | | | **average PCr/γATP over 3 years** | | |
|  | ***n*** | **Med (IQD)** | ***n*** | **Med (IQD)** | **SDM** | **SRM** | ***n*** | **Med (IQD)** | **SDM** | **SRM** | ***n*** | **Med (IQD)** | **SDM** | **SRM** | ***n*** | **Med (IQD)** | **SDM** | **SRM** |
| controls | 9 | 4.78 (4.36-4.98) |  | | | | | | | | | | | | | | | |
| patients: A | 19 | 3.45 (3.25-3.89)^**^ | 11 | 3.40 (2.95-3.93) | **-4.2** | -0.4 | 16 | 3.46 (3.22-3.67) | **-3.4** | 0.2 | 15 | 3.86 (3.18-4.15) | **-1.3** | 0.2 | 19 | 3.59 (3.18-3.78) | **-1.5** | -0.1 |
| **group** | **P_i,tot_/γATP BL** | | **P_i,tot_/γATP at Y1** | | | | **P_i,tot_/γATP at Y2** | | | | **P_i,tot_/γATP at Y3** | | | | | **average P_i,tot_/γATP over 3 years** | | |
|  | ***n*** | **Med (IQD)** | ***n*** | **Med (IQD)** | **SDM** | **SRM** | ***n*** | **Med (IQD)** | **SDM** | **SRM** | ***n*** | **Med (IQD)** | **SDM** | **SRM** | ***n*** | **Med (IQD)** | **SDM** | **SRM** |
| controls | 9 | 0.54 (0.44-0.57) |  | | | | | | | | | | | | | | | |
| patients: A | 19 | 0.77 (0.62-0.86)^**^ | 11 | 0.72 (0.69-0.94) | **1.8** | 0.3 | 16 | 0.77 (0.71-1.00) | **2.1** | 0.5 | 15 | 0.77 (0.68-0.87) | **2.0** | 0.5 | 19 | 0.76 ( 0.68-0.99) | **2.2** | 0.7 |
| **group** | **P_i,tot_/PCr BL** | | **P_i,tot_/PCr at Y1** | | | | **P_i,tot_/PCr at Y2** | | | | **P_i,tot_/PCr at Y3** | | | | | **average P_i,tot_/PCr over 3 years** | | |
|  | ***n*** | **Med (IQD)** | ***n*** | **Med (IQD)** | **SDM** | **SRM** | ***n*** | **Med (IQD)** | **SDM** | **SRM** | ***n*** | **Med (IQD)** | **SDM** | **SRM** | ***n*** | **Med (IQD)** | **SDM** | **SRM** |
| controls | 9 | 0.11 (0.10-0.13) |  | | | | | | | | | | | | | | | |
| patients: A | 19 | 0.24 (0.16-0.25)^**^ | 11 | 0.23 (0.19-0.28) | **2.2** | 0.5 | 16 | 0.22 (0.20-0.29) | **3.8** | 0.5 | 15 | 0.22 (0.16-0.27) | **2.9** | 0.2 | 19 | 0.24 (0.18-0.28) | **3.5** | 0.7 |
| **group** | **P_i,b_/P_i,tot_ BL** | | **P_i,b_/P_i,tot_ at Y1** | | | | **P_i,b_/P_i,tot_ at Y2** | | | | **P_i,b_/P_i,tot_ at Y3** | | | | | **average P_i,b_/P_i,tot_ over 3 years** | | |
|  | ***n*** | **Med (IQD)** | ***n*** | **Med (IQD)** | **SDM** | **SRM** | ***n*** | **Med (IQD)** | **SDM** | **SRM** | ***n*** | **Med (IQD)** | **SDM** | **SRM** | ***n*** | **Med (IQD)** | **SDM** | **SRM** |
| controls | 9 | 0.10 (0.04-0.14) |  | | | | | | | | | | | | | | | |
| patients: A | 18 | 0.15 (0.13-0.22)^**^ | 11 | 0.14 (0.13-0.15) | 0.3 | -0.4 | 16 | 0.13 (0.11-0.19) | **1.1** | 0.0 | 15 | 0.16 (0.13-0.28) | **1.2** | 0.5 | 18 | 0.15 (0.13-0.21) | **1.4** | 0.4 |
| **group** | **[Mg^2+^] (mM) BL** | | **[Mg^2+^] (mM) at Y1** | | | | **[Mg^2+^] (mM) at Y2** | | | | **[Mg^2+^] (mM) at Y3** | | | | | **average [Mg^2+^] (mM) over 3 years** | | |
|  | ***n*** | **Med (IQD)** | ***n*** | **Med (IQD)** | **SDM** | **SRM** | ***n*** | **Med (IQD)** | **SDM** | **SRM** | ***n*** | **Med (IQD)** | **SDM** | **SRM** | ***n*** | **Med (IQD)** | **SDM** | **SRM** |
| controls | 9 | 0.57 (0.54-0.59) |  | | | | | | | | | | | | | | | |
| patients: A | 18 | 0.50 (0.34-0.53)^*^ | 11 | 0.49 (0.47-0.58) | -0.6 | 0.4 | 15 | 0.45 (0.41-0.53) | **-1.3** | -0.4 | 13 | 0.53 (0.48-0.58) | -0.6 | 0.3 | 18 | 0.50 (0.43-0.53) | -0.5 | 0.3 |

Between controls and patients: **P*<0.006; ***P*<0.001

γATP = adenosine diphosphate (γ-resonance in ^31^P MR spectrum); BL = baseline; IQD = interquartile distance; MED = median; [Mg^2+^] = intramuscular magnesium concentration; *n* = number of patients; PCr = phosphocreatine; PDE = phosphodiesters; pH_w_ = weighted pH; P_i,b_ = alkaline inorganic phosphate; P_i,tot_ = total inorganic phosphate; PME = phosphomonoesters; SDM = standardized difference mean; SRM = standardized response mean; Y1 = year-1; Y2 = year-2; Y3 = year-3

**Supplementary Table 11 Linear Mixed Model analysis for ^31^P MRS data**

|  | **β** | **SE (β)** | **95% CI** | ***P*** |
| --- | --- | --- | --- | --- |
| **pH_w_** |  |  |  |  |
| Intercept | 7.07 | 0.02 | [7.04-7.11] | **<0.001** |
| Time=Y1 | -0.01 | 0.01 | [-0.04-0.02] | 0.363 |
| Time=Y2 | -0.01 | 0.01 | [-0.03-0.01] | 0.489 |
| Time=Y3 | -0.001 | 0.01 | [-0.02-0.02] | 0.947 |
| Site=Paris | 0.04 | 0.02 | [-0.001-0.08] | 0.051 |
| Years since onset symptoms | -0.003 | 0.001 | [-0.006- -0.0003] | 0.048 |
| FF | 0.003 | 0.001 | [0.002-0.004] | **<0.001** |
| **P_i,tot_/PCr** |  |  |  |  |
| Intercept | 0.30 | 0.02 | [0.25-0.35] | **<0.001** |
| Time=Y1 | 0.02 | 0.02 | [-0.01-0.05] | 0.236 |
| Time=Y2 | 0.06 | 0.01 | [0.03-0.09] | **<0.001** |
| Time=Y3 | 0.06 | 0.01 | [0.04-0.09] | **<0.001** |
| Site=Paris | -0.03 | 0.03 | [-0.09-0.03] | 0.353 |
| Years since onset symptoms | -0.007 | 0.002 | [-0.01- -0.002] | **0.005** |
| FF | -0.001 | 0.001 | [-0.002-0.001] | 0.470 |
| **PCr/γATP** |  |  |  |  |
| Intercept | 3.30 | 0.17 | [2.95-3.66] | **<0.001** |
| Time=Y1 | -0.12 | 0.18 | [-0.49-0.24] | 0.495 |
| Time=Y2 | -0.09 | 0.17 | [-0.42-0.25] | 0.609 |
| Time=Y3 | 0.02 | 0.16 | [-0.30-0.35] | 0.882 |
| Site=Paris | 0.38 | 0.18 | [0.003-0.75] | 0.049 |
| Years since onset symptoms | 0.05 | 0.01 | [0.02-0.08] | **0.002** |
| FF | -0.01 | 0.01 | [-0.03- -0.003] | 0.014 |
| **P_i,tot_/γATP** |  |  |  |  |
| Intercept | 0.94 | 0.08 | [0.77-1.11] | **<0.001** |
| Time=Y1 | 0.03 | 0.08 | [-0.14-0.19] | 0.754 |
| Time=Y2 | 0.17 | 0.07 | [0.02-0.32] | 0.024 |
| Time=Y3 | 0.21 | 0.07 | [0.08-0.35] | **0.004** |
| Site=Paris | -0.04 | 0.09 | [-0.22-0.14] | 0.651 |
| Years since onset symptoms | -0.02 | 0.007 | [-0.03- -0.004] | 0.014 |
| FF | 0.001 | 0.003 | [-0.005- 0.006] | 0.772 |
| **PDE/γATP** |  |  |  |  |
| Intercept | 0.43 | 0.04 | [0.36-0.51] | **<0.001** |
| Time=Y1 | -0.007 | 0.03 | [-0.07-0.06] | 0.824 |
| Time=Y2 | -0.008 | 0.03 | [-0.06-0.05] | 0.776 |
| Time=Y3 | 0.05 | 0.03 | [0.001-0.11] | 0.047 |
| Site=Paris | 0.02 | 0.04 | [-0.06-0.11] | 0.626 |
| Years since onset symptoms | -0.004 | 0.003 | [-0.01-0.003] | 0.241 |
| FF | 0.004 | 0.001 | [0.001-0.006] | **0.005** |
|  | **β** | **SE (β)** | **95% CI** | ***P*** |
| **PME/γATP** |  |  |  |  |
| Intercept | 0.33 | 0.06 | [0.21-0.45] | **<0.001** |
| Time=Y1 | 0.04 | 0.06 | [-0.07-0.15] | 0.494 |
| Time=Y2 | 0.08 | 0.05 | [-0.02-0.019] | 0.110 |
| Time=Y3 | 0.06 | 0.05 | [-0.03-0.16] | 0.204 |
| Site=Paris | -0.005 | 0.06 | [-0.13-0.12] | 0.940 |
| Years since onset symptoms | -0.007 | 0.005 | [-0.02-0.003] | 0.140 |
| FF | 0.006 | 0.002 | [0.002-0.01] | **0.005** |
| **P_i,b_/P_i,tot_** |  |  |  |  |
| Intercept | 0.19 | 0.03 | [0.13-0.26] | **<0.001** |
| Time=Y1 | -0.03 | 0.02 | [-0.07-0.01] | 0.181 |
| Time=Y2 | -0.02 | 0.02 | [-0.07-0.03] | 0.456 |
| Time=Y3 | 0.02 | 0.03 | [-0.04-0.07] | 0.489 |
| Site=Paris | 0.05 | 0.03 | [-0.01-0.12] | 0.118 |
| Years since onset symptoms | -0.007 | 0.002 | [-0.01-0.002] | **0.006** |
| FF | 0.003 | 0.001 | [0.002-0.006] | **0.001** |
| **[Mg^2+^] (mM)** |  |  |  |  |
| Intercept | 0.47 | 0.03 | [0.42-0.53] | **<0.001** |
| Time=Y1 | 0.06 | 0.04 | [-0.02-0.14] | 0.181 |
| Time=Y2 | 0.04 | 0.03 | [-0.02-0.10] | 0.456 |
| Time=Y3 | 0.09 | 0.04 | [0.02-0.17] | 0.489 |
| Site=Paris | 0.05 | 0.03 | [-0.01-0.12] | 0.118 |
| Years since onset symptoms | 0.004 | 0.002 | [0.0002-0.007] | 0.039 |
| FF | -0.004 | 0.001 | [-0.006- -0.003] | **<0.001** |

^b^Estimate for site=Newcastle at baseline

γATP = adenosine diphosphate (γ-resonance in ^31^P MR spectrum); CI = confidence interval; FF = fat fraction (in %); [Mg^2+^] = intramuscular magnesium concentration (in mM); PCr = phosphocreatine; PDE = phosphodiesters; pH_w_ = weighted pH; P_i,b_ = alkaline inorganic phosphate; P_i,tot_ = total inorganic phosphate; PME = phosphomonoesters; SE = standard error; Y1 = year-1; Y2 = year-2; Y3 = year-3

**Supplementary Table 12 Correlation analysis of global segment quantitative MRI indices, functional and clinical parameters**

|  | Thigh FF BL (%) | Leg FF BL (%) | Thigh cCSA BL  (cm^2^) | Leg cCSA BL  (cm^2^) | Thigh water T_2_ BL (ms) | Leg water T_2_  BL (ms) | Thigh ΔFF after Y3 (%) | Leg ΔFF after Y3 (%) | Thigh ΔcCSA after Y3 (%) | Leg ΔcCSA after Y3 (%) | total NSAD after Y3 | ΔNSAD after Y3 | CK  BL  (U/L) | Age  BL  (years) | Years since symptom onset BL (years) |
| --- | --- | --- | --- | --- | --- | --- | --- | --- | --- | --- | --- | --- | --- | --- | --- |
| MRI |  |  |  |  |  |  |  |  |  |  |  |  |  |  |  |
| Leg FF BL (%) | **0.84^**^** |  |  |  |  |  |  |  |  |  |  |  |  |  |  |
| Thigh cCSA BL (cm^2^) | **-0.85^**^** | **-0.67^**^** |  |  |  |  |  |  |  |  |  |  |  |  |  |
| Leg cCSA BL (cm^2^) | **-0.63^**^** | **-0.74^**^** | **0.77^**^** |  |  |  |  |  |  |  |  |  |  |  |  |
| Thigh water T_2_ BL (ms) | **-0.30^*^** | -0.26 | 0.14 | -0.03 |  |  |  |  |  |  |  |  |  |  |  |
| Leg water T_2_ BL (ms) | -0.26**^*^** | -0.19 | **0.34^*^** | 0.20 | **0.49^*^** |  |  |  |  |  |  |  |  |  |  |
| Thigh ΔFF after Y3 (%) | -0.27 | -0.16 | 0.19 | 0.06 | **0.52^**^** | **0.29^*^** |  |  |  |  |  |  |  |  |  |
| Leg ΔFF after Y3 (%) | **-0.39^*^** | **-0.46^**^** | **0.33^*^** | **0.47^*^** | **0.33^*^** | 0.26 | **0.43^**^** |  |  |  |  |  |  |  |  |
| Thigh ΔcCSA after Y3 (%) | **0.48^*^** | 0.39 | **-0.55^**^** | -0.24 | **-0.37^*^** | -0.22 | **-0.63^**^** | **-0.30^*^** |  |  |  |  |  |  |  |
| Leg ΔcCSA after Y3 (%) | **0.32^*^** | **0.50^**^** | **-0.34^*^** | **-0.58^**^** | -0.16 | -0.02 | -0.26 | **-0.69^**^** | **0.42^**^** |  |  |  |  |  |  |
| FUNCTIONAL/CLINICAL |  |  |  |  |  |  |  |  |  |  |  |  |  |  |  |
| Total NSAD after Y3 | **-0.76^**^** | **-0.67^**^** | **0.84^**^** | **0.66^**^** | 0.34 | 0.23 | 0.02 | 0.22 | -0.36 | -0.21 |  |  |  |  |  |
| ΔNSAD after Y3 | 0.15 | -0.13 | -0.22 | 0.16 | -0.43 | -0.42 | -**0.55^*^** | -0.48 | 0.42 | 0.08 | 0.22 |  |  |  |  |
| CK BL (U/L)^a^ | **-0.50^**^** | -**0.54^**^** | **0.41^*^** | 0.35 | **0.48^*^** | **0.38^*^** | **0.38^*^** | 0.41 | -0.37 | -0.08 | 0.17 | -0.47 |  |  |  |
| Age BL (years) | **0.42^**^** | **0.48^**^** | **-0.29^*^** | -0.21 | **-0.57^**^** | **-0.33^*^** | **-0.29^*^** | -**0.31^*^** | **0.40^**^** | 0.18 | -0.14 | 0.39 | **-0.75^**^** |  |  |
| Years since symptom onset BL (years) | **0.59^**^** | **0.51^**^** | **-0.48^*^** | **-0.48^**^** | **-0.39^*^** | **-0.40^**^** | **-0.33^*^** | **-0.52^**^** | **0.33^*^** | **0.33^*^** | **-0.38^*^** | 0.42 | **-0.70^**^** | **0.73^**^** |  |
| BMI BL | **0.33^*^** | **0.36^*^** | -0.07 | -0.005 | -0.15 | 0.10 | -0.14 | -0.09 | 0.06 | 0.13 | -0.13 | 0.07 | 0.08 | 0.10 | 0.07 |

*<0.017;**<0.001

BL = baseline; CK = creatine kinase; cCSA = contractile cross-sectional area; NSAD = North Star Assessment for limb-girdle muscular Dystrophies; FF= fat fraction; Y3 = year-3; ΔcCSA = change in cCSA (in %, relative); ΔFF = change in FF (in %, absolute); ΔNSAD = change in NSAD

**Supplementary Table 13 Correlation analysis of ^31^P MRS indices and quantitative MRI in anterior leg compartment, and functional and clinical parameters**

|  | pH_w_  BL | PDE/  γATP  BL | PME/  γATP  BL | PCr/  γATP  BL | P_i,tot_/  γATP  BL | P_i,tot_/  PCr  BL | P_i,b_/  P_i,tot_  BL | [Mg^2+^]  BL (mM) | FF BL (%) | water T_2_ BL  (ms) | ΔFF after Y3 (%) | ΔcCSA after Y3 (%) | MMT BL | ΔNSAD after Y3 | CK BL (U/L) | Age BL (years) | Years since symptom onset BL (years) |
| --- | --- | --- | --- | --- | --- | --- | --- | --- | --- | --- | --- | --- | --- | --- | --- | --- | --- |
| ^31^P MRS/MRI |  |  |  |  |  |  |  |  |  |  |  |  |  |  |  |  |  |
| PDE/γATP BL | 0.40 |  |  |  |  |  |  |  |  |  |  |  |  |  |  |  |  |
| PME/γATP BL | **0.63^*^** | **0.67^*^** |  |  |  |  |  |  |  |  |  |  |  |  |  |  |  |
| PCr/γATP BL | **-0.68^**^** | -0.26 | -0.39 |  |  |  |  |  |  |  |  |  |  |  |  |  |  |
| P_i,tot_/γATP BL | 0.35 | 0.23 | 0.44 | -0.53 |  |  |  |  |  |  |  |  |  |  |  |  |  |
| P_i,tot_/PCr BL | **0.54^*^** | 0.26 | 0.45 | **-0.82^**^** | **0.88^**^** |  |  |  |  |  |  |  |  |  |  |  |  |
| P_i,b_/P_i,tot_ BL | 0.58 | 0.38 | 0.54 | -0.57 | 0.38 | 0.51 |  |  |  |  |  |  |  |  |  |  |  |
| [Mg^2+^] BL (mM) | **-0.60^*^** | -0.08 | -0.39 | **0.70^*^** | -0.53 | **-0.74^*^** | -0.60 |  |  |  |  |  |  |  |  |  |  |
| FF BL (%) | **0.73^**^** | **0.66^*^** | **0.71^**^** | -0.41 | 0.19 | 0.32 | 0.54 | -0.48 |  |  |  |  |  |  |  |  |  |
| water T_2_ BL (ms) | 0.46 | -0.05 | 0.36 | **-0.63^*^** | 0.47 | **0.69^*^** | 0.17 | -0.59 | 0.18 |  |  |  |  |  |  |  |  |
| ΔFF after Y3 (%) | 0.21 | 0.40 | 0.49 | -0.41 | **0.70^*^** | 0.60 | 0.17 | -0.22 | 0.26 | 0.35 |  |  |  |  |  |  |  |
| ΔcCSA after Y3 (%) | -0.08 | -0.15 | -0.32 | -0.01 | -0.55 | -0.28 | 0.03 | -0.07 | 0.01 | -0.02 | **-0.69^*^** |  |  |  |  |  |  |
| FUNCTIONAL/CLINICAL |  |  |  |  |  |  |  |  |  |  |  |  |  |  |  |  |  |
| MMT BL | **-0.65^*^** | -0.05 | -0.28 | 0.40 | -0.42 | -0.42 | **-**0.46 | 0.42 | -0.35 | -0.19 | 0.55 | 0.59 |  |  |  |  |  |
| ΔNSAD after Y3 | -0.27 | -0.41 | -0.70 | 0.48 | -0.55 | -0.65 | -0.40 | 0.27 | -0.47 | -0.70 | **-0.79^*^** | 0.48 | 0.19 |  |  |  |  |
| CK BL (U/L) | 0.59 | -0.08 | 0.39 | **-0.69^*^** | 0.53 | **0.72^*^** | 0.40 | -0.57 | 0.14 | **0.69^*^** | 0.27 | -0.44 | -0.49 | -0.36 |  |  |  |
| Age BL (years) | -0.18 | 0.01 | -0.20 | 0.49 | -0.36 | -0.51 | -0.35 | 0.33 | 0.04 | -0.53 | -0.15 | 0.06 | -0.04 | 0.35 | **-0.70^*^** |  |  |
| Years since onset symptoms BL (yrs) | -0.02 | 0.01 | -0.13 | 0.52 | -0.52 | **-0.68^*^** | -0.29 | 0.63 | 0.05 | 0.48 | -0.24 | -0.13 | -0.10 | 0.37 | -0.49 | **0.60^*^** |  |
| BMI BL | 0.39 | 0.01 | 0.17 | -0.20 | 0.41 | 0.28 | 0.15 | -0.05 | 0.14 | -0.13 | -0.32 | 0.55 | -0.53 | -0.11 | 0.53 | 0.04 | 0.21 |

*<0.006;**<0.001

γATP = adenosine diphosphate (γ-resonance in ^31^P MR spectrum); BL = baseline; CK = creatine kinase; FF = fat fraction (in %); MMT = manual muscle testing; [Mg^2+^] = intramuscular magnesium concentration; PCr = phosphocreatine; PDE = phosphodiesters; pH_w_ = weighted pH; P_i,b_ = alkaline inorganic phosphate; P_i,tot_ = total inorganic phosphate; PME = phosphomonoesters; Y3 = year-3; ΔcCSA = change in cCSA (in %, relative); ΔFF = change in FF (in %, absolute); ΔNSAD = change in NSAD
